# Ellagic Acid and Cancer Hallmarks: Insights from Experimental Evidence

**DOI:** 10.3390/biom13111653

**Published:** 2023-11-15

**Authors:** Martina Čižmáriková, Radka Michalková, Ladislav Mirossay, Gabriela Mojžišová, Martina Zigová, Annamária Bardelčíková, Ján Mojžiš

**Affiliations:** 1 Department of Pharmacology, Faculty of Medicine, Pavol Jozef Šafárik University, 040 01 Košice, Slovakia; martina.cizmarikova@upjs.sk (M.Č.); radka.michalkova@upjs.sk (R.M.); chripkova.martina@gmail.com (M.Z.); annamaria.bardelcikova@upjs.sk (A.B.); 2 Center of Clinical and Preclinical Research MEDIPARK, Faculty of Medicine, Pavol Jozef Šafárik University, 040 01 Košice, Slovakia; gabriela.mojzisova@upjs.sk

**Keywords:** cancer, hallmarks, ellagic acid, chemoprevention, chemotherapy

## Abstract

Cancer is a complex and multifaceted disease with a high global incidence and mortality rate. Although cancer therapy has evolved significantly over the years, numerous challenges persist on the path to effectively combating this multifaceted disease. Natural compounds derived from plants, fungi, or marine organisms have garnered considerable attention as potential therapeutic agents in the field of cancer research. Ellagic acid (EA), a natural polyphenolic compound found in various fruits and nuts, has emerged as a potential cancer prevention and treatment agent. This review summarizes the experimental evidence supporting the role of EA in targeting key hallmarks of cancer, including proliferation, angiogenesis, apoptosis evasion, immune evasion, inflammation, genomic instability, and more. We discuss the molecular mechanisms by which EA modulates signaling pathways and molecular targets involved in these cancer hallmarks, based on in vitro and in vivo studies. The multifaceted actions of EA make it a promising candidate for cancer prevention and therapy. Understanding its impact on cancer biology can pave the way for developing novel strategies to combat this complex disease.

## 1. Introduction

According to the WHO, cancer is the second leading cause of death globally, following cardiovascular diseases [1]. Furthermore, the Global Cancer Observatory (GLOBOCAN) reported that more than 19 million new cases and approximately 10 million deaths were associated with cancer worldwide in 2020. In addition, predictions for the future indicate that by the year 2040, the global population could experience a surge in cancer incidence reaching 28.4 million cases [2].

Cancer is a complex and multifaceted disease with many different types, each with its own distinct characteristics and behaviors, and is generally characterized by the uncontrolled and abnormal growth of cells within the body [3]. Cancer risk factors include genetics, exposure to carcinogens, lifestyle factors, and certain infections [4].

In contrast to normal cells, which adhere to stringent regulations governing their growth and replication, cancer cells circumvent these checks and possess the capability to not only infiltrate adjacent tissues but also disperse to distant locations within the body via the bloodstream or lymphatic system [5].

## 2. Cancer Hallmarks

Cancer cells display a series of unique characteristics that differentiate them from normal cells. These characteristics collectively play a role in the unrestrained proliferation and invasive nature that define cancer. These traits are commonly referred to as “cancer hallmarks”.

**Figure 1:**
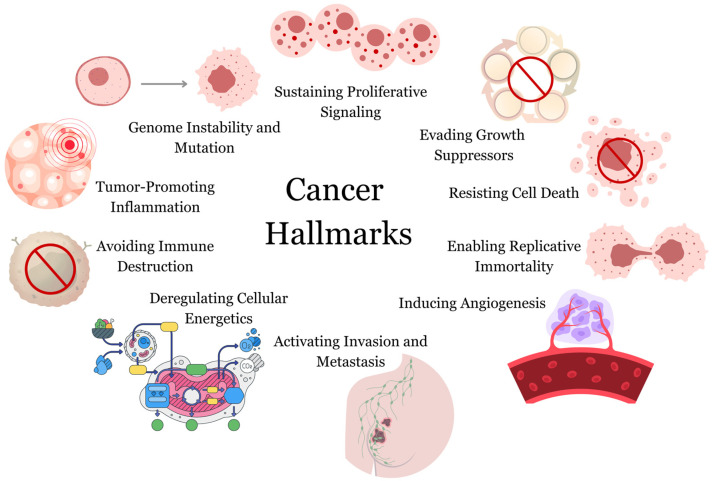
The Hallmarks of Cancer. This diagram illustrates the hallmark traits that are typically possessed by the majority of cancers.

More than two decades ago, Hanahan and Weinberg [6] published a seminal review known as “The Hallmarks of Cancer” to provide structure to the intricate landscape of cancer biology. In this publication, they sought to categorize the complex facets of cancer into six key hallmarks. A decade later, in a subsequent review [7], the original six hallmarks were expanded to eight, with two enabling characteristics (tumor-promoting inflammation and genome instability and mutation). However, twelve years later, it has become clear that these newer additions, similar to the original six hallmarks, can now be regarded as fundamental characteristics of cancer. They are now recognized and incorporated as such into the current representation of cancer hallmarks (Figure 1).

*Sustaining Proliferative Signaling*: Cancer cells can receive and send signals that stimulate their own uncontrolled growth and division, even without external growth signals.

*Evading Growth Suppressors*: Cancer cells can bypass the mechanisms that normally inhibit their growth, such as checkpoints that prevent damaged cells from proliferating.

*Resisting Cell Death*: Cancer cells can evade programmed cell death (apoptosis), which is a mechanism that eliminates damaged or abnormal cells.

*Deregulating Cellular Energetics*: Cancer cells can rewire their metabolic pathways to support their high energy needs for rapid growth and division.

*Enabling Replicative Immortality*: Cancer cells can maintain their ability to divide indefinitely by avoiding the usual limitations on the number of divisions that normal cells experience.

*Inducing Angiogenesis*: Cancer cells can stimulate the growth of new blood vessels (angiogenesis) to provide them with the necessary nutrients and oxygen for continued growth and survival.

*Activating Invasion and Metastasis*: Cancer cells can invade nearby tissues and spread to distant sites in the body, a process known as metastasis, which is a major cause of cancer-related deaths.

*Avoiding Immune Destruction*: Cancer cells can evade recognition and attack by the immune system, allowing them to escape immune surveillance.

*Tumor-Promoting Inflammation*: Chronic inflammation in the tumor microenvironment can promote cancer development by creating a favorable environment for cancer cells to grow and evade the immune system.

*Genome Instability and Mutation*: Cancer cells often exhibit high levels of genetic instability and mutations, which contribute to the diversity of cancer cell populations and their ability to adapt to different conditions.

These hallmarks are not exclusive; they often interact with and reinforce each other to drive the complex process of cancer development. Understanding these fundamental traits has led to the development of targeted therapies that aim to interfere with specific hallmark features, offering new strategies for cancer treatment. However, it is important to note that each cancer type and even individual tumors can exhibit variations in the prominence of these hallmarks, making each case unique and challenging to treat effectively.

## 3. Ellagic Acid

Ellagic acid (EA) is a naturally derived polyphenol present in a variety of fruits, nuts, and vegetables. Notably concentrated in fruits such as pomegranates, strawberries, raspberries, blackberries, and nuts such as walnuts. This compound also shows higher levels in certain tree species’ wood and bark, including *Quercus* spp., *Eucalyptus* spp., and *Castanea* spp. [8].

In addition to the naturally occurring unbound EA found in plant-based foods, a substantial portion of this compound is produced within the gastrointestinal tract of both humans and animals. This production is a result of the enzymatic or non-enzymatic breakdown of dietary polyphenolic molecules called ellagitannins [9]. Within the gastrointestinal tract, EA has restricted bioavailability, primarily due to its hydrophobic nature and very low water solubility. Consequently, only a fraction of EA is absorbed in the small intestine. The unabsorbed EA molecules undergo further metabolic transformations within the large intestine, facilitated by intestinal microorganisms. This process leads to the formation of a group of lipophilic metabolites known as urolithins, which are subsequently absorbed into the bloodstream [10]. Readers can find more about the fate and biological effects of EA in a recent article authored by Sharifi-Rad and colleagues [11].

EA has gained attention in the last decades due to its broad range of biological effects and potential health benefits. It is known for its strong antioxidant properties [12,13], anti-inflammatory effects [14,15], and antimicrobial [16,17,18] and antimutagenic effects [19]. Furthermore, many studies have reported cardioprotective [20], neuroprotective [21,22], gastroprotective [23], hepatoprotective [24], and nephroprotective [25] effects of EA.

**Figure 2:**
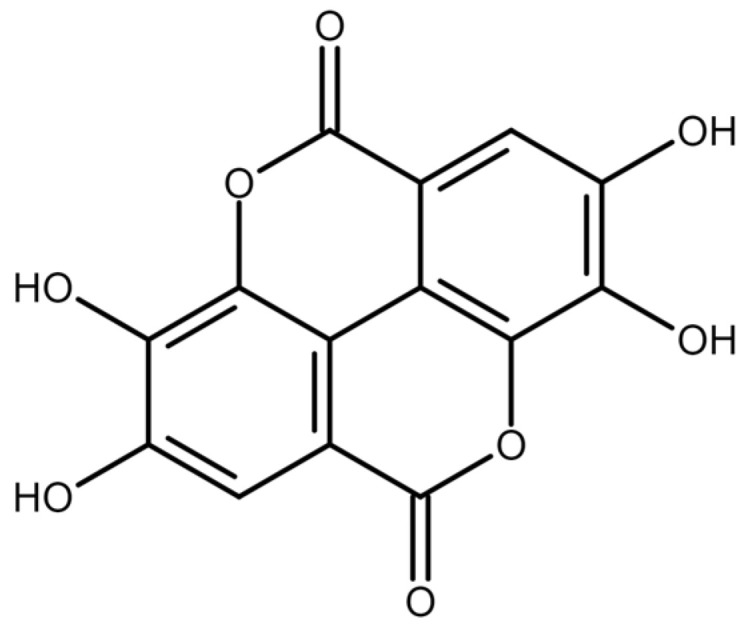
Chemical structure of ellagic acid.

Moreover, numerous studies indicate that EA possesses properties that can inhibit cell proliferation and potentially counteract cancer development. In light of this perspective, the aim of this review is to clarify the mechanisms by which EA impacts fundamental cancer characteristics. The chemical structure of EA is shown in Figure 2.

### 3.1. Ellagic Acid and Sustaining Proliferative Signaling

One of the fundamental hallmarks of cancer is sustained (uncontrolled chronic cell proliferation) [7], often arising from deregulations in the normal cell cycle [26]. Generally, gap (G) phases represent the essential cell cycle checkpoints, in which the cell cycle is slowed down or stopped to ensure the accuracy and integrity of cell division. The importance of these checkpoints lies in their ability to maintain genomic stability and prevent the proliferation of damaged or mutated cells. Growing evidence supports the view that the main restriction checkpoint is G1-S transition [27], and the complex of cyclin D with two cyclin-dependent kinases (CDK4 and CDK6) seems to be its primary positive regulatory element [28]. The primary function of this complex is to sustain the phosphorylation of the retinoblastoma protein (pRB), ultimately leading to the progression of the cell cycle. On the other hand, the dephosphorylated state of this protein is widely recognized as a tumor growth suppressor, as it halts the cell cycle through the pRB pathway. Moreover, DNA damage signaling is the primary hindrance to entry into the S phase of the cell cycle.

Generally, CDK4/6 activity is modulated via the association with D-type cyclins (D1, D2, D3), which activates it, and via the binding of cyclin-dependent kinase inhibitors (CKIs) from the INK4 family (p16, p15, p18, p19), which inhibits it, ensuring proper control of the cell cycle [29]. However, additional kinases and CDKs can be involved in cell cycle control at other checkpoint sites [30].

Previous data indicate that the sustained proliferation in cancer cells may be attributed to various factors, including but not limited to altered expression or activity of molecules regulating the cell cycle checkpoints and the constitutive activation of various signal transduction pathways [26]. In brief, these mechanisms include increased generation of ligands (e.g., epidermal growth factor, EGF) for growth factor receptors (GFRs), increased receptor signaling as a result of upregulated GFRs at the surface of cancer cells, receptor activation without the need for ligands (ligand-independent firing), and constitutive activation of the elements within signaling pathways. However, mitogenic signaling in cancer cells is generally better comprehended than the action of mitogenic factors themselves. Furthermore, the signaling molecules (e.g., protein-kinases) of the pathways regulating the cell cycle progression have pivotal roles in various other processes that are important in oncogenesis (e.g., apoptosis, cell differentiation, angiogenesis, cell migration, and adhesion or stress response) [31]. Consequently, studying these pathways in cancer development presents considerable challenges.

Interestingly, signaling pathways that influence the G1 phase progression of the cell cycle typically do not control CDK levels directly. Instead, they primarily impact CDK activity by regulating the expression of their associated cyclin partners and a set of CKIs [27]. In the control of the cell cycle, cyclin D fulfills its role in a complex with specific CDKs (CDK4 and CDK6), acting as their allosteric modulator. The newly established complex can interact with CDK-activating kinases (CAKs), initiating phosphorylation and subsequent activation of CDKs [32]. Referring to the prior text in the article, the active cyclin D-CDK4/CDK6 complex is a pivotal regulator of pRB phosphorylation [28]. The literature on cyclin D also reported its additional functions, such as the sequestration of two important CKIs p21 and p27 [33], phosphorylation of other transcription factors involved in the regulation of the cell cycle Smad3 and forkhead box M1 (FOXM1) [34], effects on mitochondrial metabolism [35], and the effects in the cellular response to genotoxicity [36]. The available evidence seems to suggest that transcription and activity of cyclin D are significantly reliant on the mitogenic signals from three key signaling pathways: the MAPK pathway (Ras/Raf/MEK-ERK for cyclin D synthesis), the PI3K/Akt pathway (for cyclin D stability), and the Wnt/ß-catenin pathway (ß-catenin/TCF-LEF) [37]. Moreover, the transcription of cyclin D can also be regulated by other signals, such as the Notch pathway, extracellular matrix, cytokines (through STAT3), and immune signals (through nuclear factor kappa B, NF-κB) [27]. Based on the literature, there are three distinct types of D-type cyclins (cyclin D1–3), each of which is encoded by the genes *CCND1*, *CCND2*, and *CCND3*, respectively [38]. All three members of this family can interact with CDK4 and CDK6, playing important roles in the regulation of the cell cycle. In addition to their presence in various healthy cells, they have also been identified in tumor cells. However, their levels may differ depending on the particular tissue and type of cancer. Next, it has been reported that their oncogenic potential is typically linked to genetic deregulation, often stemming from gene amplification, mutations, or other genetic alterations [37]. Interestingly, the amplification of *CCND1* was found to be the most frequently observed genetic event in cancer [39].

Another key player in the narrative of sustaining proliferative signaling is the Myc protein, encoded by the c-Myc gene. Abnormalities in Myc expression or function are closely linked to various cancers [40]. Myc operates as a transcription factor, wielding control over a multitude of target genes involved in cell cycle progression, cell proliferation, differentiation, and apoptosis [41]. Its pivotal role lies in promoting the transition from G1 to S phase by upregulating cyclin D2 expression and inhibiting the expression of genes encoding CKIs such as p21 and p15. Furthermore, Myc can influence other crucial cell cycle regulators, including CDK4, E2F1, and CDC25A, while downregulating p27 [40]. The regulation of the Myc protein is a complex process involving multiple critical signaling pathways, such as Notch1 [42,43], MAPK [44], PI3K/Akt/GSK-3β [45], Wnt-ß-catenin [46], Janus kinase 2, and signal transducer and activator of transcription 3 (JAK-STAT3) [47] or Hedgehog [48].

Beyond cyclins and Myc, emerging research has shed light on the role of mini-chromosome maintenance (MCM) proteins in cell cycle checkpoints [49]. MCM3, in particular, has been implicated in regulating the cell cycle, with its knockdown leading to reduced expression of key cell cycle progression elements and G1/S transition suppression [50]. Elevated expression of MCM2, MCM5, and MCM7 has also been identified as potential prognostic biomarkers associated with unfavorable outcomes in several cancer types [51].

Classical isoenzymes of the protein kinase C (PKC) family can also be involved in cell cycle regulation and cell growth by influencing its downstream targets such as Myc, cyclins, CDKs, CKIs [52,53], or NF-κB [54].

In addition, COX-2 may contribute to the modulation of the cell cycle control, e.g., via inhibition of cyclin D generation [55].

Based on the above, the inhibition of sustaining proliferative signaling pathways represents an important strategy in the quest for effective cancer therapy.

**Figure 3:**
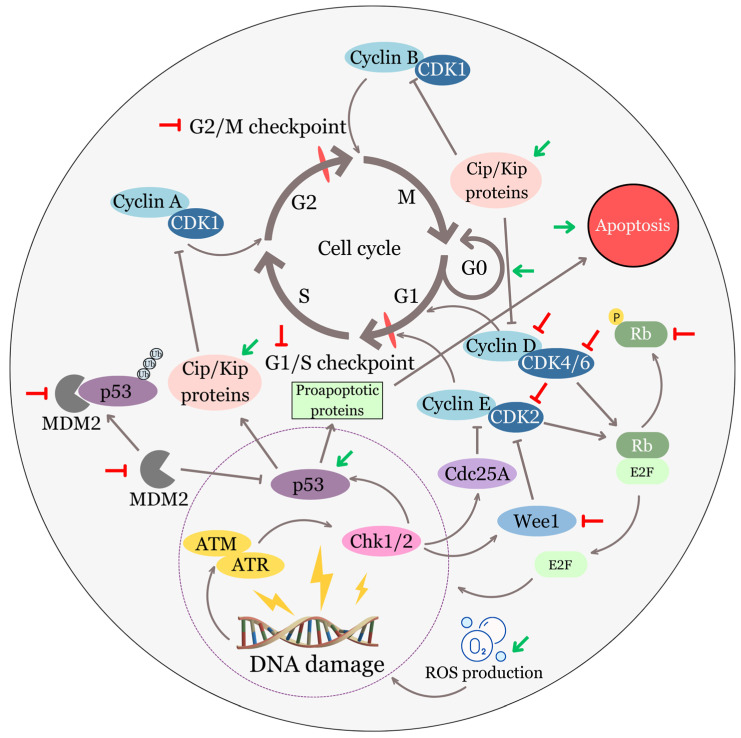
Ellagic acid eliminates self-sufficiency in growth signals and restores responsiveness to growth-inhibitory signals. Upward-pointing green arrows signify heightened expression/activation, while red T bars indicate lowered expression or reduced activity.

Accumulated evidence has confirmed that EA can attenuate several specified signal molecules or signal pathways involved in cell cycle control and proliferation in different cancer cells (Figure 3).

For instance, EA effectively suppressed cell proliferation of two human ovarian cancer cells (ES-2 and PA-1). It achieved this by causing cell cycle arrest at the G1 phase in both cell lines. This arrest was attributed to the reduction of cyclin D1 and E levels, as well as to the upregulation of p53 and p21 proteins [56].

In another study, the analysis of murine xenografts of human pancreatic cancer cells (PANC-1) highlighted that the antitumor effect of EA may manifest via the inhibition of various signaling pathways and molecules [57]. Some of the signaling pathways (Notch, PI3K/Akt, or Hedgehog) and molecules (cyclin D1, CDK2, CDK6, COX-2, IL-6, and IL-8) investigated in this study, based on the knowledge from previous research, could have an impact on cell cycle regulation to some extent. Subsequent studies have substantiated the capacity of EA to impede the proliferation of pancreatic cancer cells in laboratory settings (in vitro using PANC-1 cells) and within living organisms (in vivo, employing mice bearing PANC-1 tumors) [58]. Furthermore, in vitro analyses conducted on pancreatic cancer cells have revealed a connection between EA treatment and the arrest of the cell cycle at the G1 phase. Additionally, there has been a notable reduction in the protein expression of COX-2 and NF-κB as a result of this treatment.

Next, in vivo, experiments on U87 glioblastoma mouse xenografts showed that EA can mitigate cell proliferation by suppressing Akt and Notch signaling pathways, as well as reducing the levels of several signaling molecules, including cyclin D1, CDK2, and CDK6 [59]. In human bladder cancer cells (TSGH8301), EA demonstrated the capacity to hinder cell proliferation by concurrently suppressing the expression of two proteins governing the cell cycle, namely CDK2 and WEE1 kinase [60]. Additionally, it prompted a state of cell cycle arrest specifically at the G1 phase. Other researchers have documented a decrease in cell proliferation, a halt in the cell cycle at the G1 phase, and the induction of apoptosis in human cervical carcinoma cells (HeLa) treated with EA. This was observed concurrently with the suppression of the JAK/STAT3 pathway [61]. Furthermore, there is a suggestion that the reduced cell proliferation in HeLa cells induced via EA treatment might be associated with the decreased expression of epidermal growth factor receptor (EGFR) protein [62]. In the identical cell line, EA also demonstrated this effect when used in combination with curcumin. Moreover, the authors observed cell cycle arrest either in the S phase when EA was administered alone or in the G2/M phase when EA was combined with curcumin. As reported in the literature, hormones are other regulators of cell cycle progression via upregulation of factors promoting cell cycle progression (e.g., cyclin D1 or Cdk-activating phosphatase, Cdc25A) and downregulation of CKIs (p21, p27) expression [63,64]. Interestingly, EA acted as a natural estrogen antagonist in HeLa cells, primarily acting through ERbeta [65]. In addition, in endometrial cancer cells (KLE and AN3CA), EA has also demonstrated a reduction in cell proliferation as a result of inducing cell cycle arrest in the G1 phase, ultimately leading to apoptosis [66]. Furthermore, both bioinformatics analyses and in vitro experiments have underscored the significance of two key signaling molecules, phosphatidylinositol-4,5-bisphosphate 3-kinase catalytic subunit alpha (PIK3CA) and phosphatidylinositol-4,5-bisphosphate 3-kinase regulatory subunit 1 (PIK3R1), in the anticancer effects of EA. It has been documented that EA downregulates their gene and protein expression. While this study has examined the downregulation of these two signaling molecules in the context of inhibiting cell migration, it is important to acknowledge that these actions may potentially contribute to various other anti-tumor effects of EA as well through involvement in several signaling pathways.

EA was also found to inhibit signaling pathways that regulate cell proliferation in prostate cancer cells (PC3), even in the presence of upregulated gene expression of IL-6 [67]. It specifically downregulated the expression of phosphorylated ERK1/2, AKT, and STAT3, effectively suppressing cell proliferation.

Earlier studies have identified the downregulation of the TGF-β/Smad3 signaling pathway as a critical factor contributing to the inhibition of cell proliferation and the induction of cell cycle arrest in EA-treated MCF-7 breast cancer cells [68,69]. Recent research on human breast cancer cells (MCF-7 and MDA-MB-231) documented EA as a potent inhibitor of CDK6 [70]. The compound not only impeded tumor growth but also suppressed the gene and protein expression of CDK6 within MCF-7 and MDA-MB-231 cancer cells. Additionally, EA demonstrated the capacity to bind to CDK6 and effectively inhibit its activity.

In metastatic human melanoma cell lines (namely 1205Lu, WM852c, and A375), EA exhibited several noteworthy in vitro effects. Notably, it proficiently inhibited cell proliferation, induced cell cycle arrest at the G1 phase suppressed NF-κB activity, and significantly diminished gene expression of pro-inflammatory cytokines such as IL-1β and IL-8 [71]. Furthermore, apoptosis was also observed in the study. Later experiments performed on melanoma cancer cells (WM115 and A375) elucidated that EA can hinder cell proliferation by inhibiting the EGFR signaling pathway [72]. Specifically, significantly reduced protein expression of the phosphorylated form of EGFR was one of the obtained results. A subsequent in vivo analysis using a nude mouse A375 tumor xenograft model confirmed the ability of EA to inhibit phosphorylation of EGFR and reduce tumor size and weight.

In human colon cancer cells, specifically HT29 and HT116, EA administration was observed to notably inhibit cell proliferation and downregulate the Wnt/β-catenin signaling pathway [73]. Inhibition of this signaling cascade was linked to a reduction in the protein expression of β-catenin. Additionally, there was an elevation in the levels of phosphorylated β-catenin, axin 1, and axin 2, suggesting enhanced degradation of β-catenin. Moreover, the downstream components of this cascade, specifically Myc, cyclin D1, and survivin, exhibited decreased levels. Furthermore, a diminished CDK8 level was observed. In the next study, EA was also found to impede the proliferation of HCT-15 colon adenocarcinoma cells [74]. Subsequent analyses showed prompt cell cycle arrest (G2/M) and apoptosis induction via modulation of the PI3K/Akt signaling pathway in EA-treated HCT-15 cancer cells. In other human colorectal cancer cell lines (HCT-116 and CaCo-2), EA exhibited a significant capacity to impede cell proliferation. It also demonstrated the ability to suppress the expression of K-ras protein, along with the significant suppression of Akt phosphorylation at both Thr308 and Ser473. Moreover, EA caused cell cycle arrest at either the G1 or S phase [75]. Later, the experimental data conducted on HCT-116 cells pointed to the ability of EA to retard cell proliferation and elicit G0/G1 cell cycle arrest and apoptosis, all likely as a result of stimulation of the TGF-ß1/Smad3 signaling pathway [76]. Triggering of this pathway resulted in an upregulation of p15, a well-established CKI responsible for dampening the activity of the CDK4/CDK6 complex.

In hepatocellular carcinoma cells (HepG2), EA treatment resulted in a notable decrease in both gene and protein expression among several members of the MCM family (MCM2-7) and CDKs (CDK2 and CDK4), concomitantly with upregulated p21 and decreased phosphorylation of pRB [77]. Furthermore, cell cycle arrest in the G1 phase was observed.

The treatment with EA was also observed to be associated with the suppression of PKC signaling in Dalton’s lymphoma-bearing (DL) mice, resulting in the inhibition of cell proliferation [78]. Specifically, EA has demonstrated a notable downregulatory impact on the expression of classical isoenzymes of the PKC family (PKCα, PKCβ, and PKCγ). Moreover, EA also exhibited the capacity to diminish the overall activity of PKC and the expression of downstream molecules, including NF-kB and c-Myc. On the other hand, the expression of TGF-β1 was upregulated. According to the DrugBank database (Edmonton, AB, Canada), EA serves as a competitive inhibitor for both protein kinase C alpha and beta types [66].

### 3.2. Ellagic Acid and Evading Growth Suppressors

Insensitivity to anti-growth signals is another typical characteristic of many malignancies. It occurs when the function of growth suppressors encoded by tumor suppressor genes is deregulated. Under normal conditions, the role of tumor suppressors is to control the cell cycle and ensure the repair of DNA damage, limit inappropriate cell growth, and reduce invasive and metastatic potential. Their normal function thus prevents the development and progression of cancer or the failure of cancer treatment. However, genetic and epigenetic alterations, as well as other factors, can alter their function and subsequently they may even gain oncogenic potential [79,80]. Therefore, the complexity of molecular interactions requires further research.

Anti-growth signaling includes several important molecules [80]. However, mainly p53, pRB, p16, and p21 proteins were reported as common tumor suppressors controlling the cell cycle [81]. Based on evidence from the literature, EA may modulate specifically three of them (p53, p21, and pRB). In addition, the effects of EA on p53 and p21 have often been evaluated together in research.

Among a variety of known growth suppressors, p53, dubbed “guardian of the genome” [82], is the most studied. It is a transcription factor induced by many endogenous and exogenous stressors [83]. Its function is to regulate several types of programmed cell death [84] and pathways for the generation of reactive oxygen species (ROS) [85], but it is also involved in controlling cell cycle and DNA repair [81] besides many other functions associated with cancer progression and cancer treatment failure [79]. The protein is frequently genetically altered or absent in various cancer cells. Genetic alterations occur in more than 50% of all cancers with the highest genetic alterations frequency in small cell lung cancer, non-melanoma cancer, and ovarian cancer [84]. In this sense, downstream target genes of p53 are not activated, and the protein loses its tumor-suppressive activity. Moreover, mutated forms of p53 independently activate several signaling pathways or molecules, e.g., NF-Y/p300 [86], Y/NF-κB–MAP2K3 [87], EGFR/ERK due to suppression miR-27a [88], YAP–cyclin A/cyclin B/CDK1 [89], YAP/TEAD–circPVT1 [90], STAT3 [91] and CDK4–cyclin D1– c-MYC [79] to encourage cell proliferation. Interestingly, the crucial negative regulator of p53 is a nuclear protein known as a murine double minute clone 2 (MDM2). It functions mostly as an E3 ubiquitin ligase that targets p53 [92] and some other proteins involved in cell cycle regulation and apoptosis [93] for proteasomal degradation. However, it also blocks the activity of intact p53 by inhibiting the p53 transactivation [94]. In short, cancer development and progression can occur despite wild-type p53 as a consequence of MDM2 upregulation. The prevalence of MDM2 amplification has been reported, in various types of cancer, from 3.5% [95] to 5.5% [96], mostly in sarcomas [97]. Moreover, MDM2 amplification may be associated with drug resistance [98]. Recently, several MDM2 inhibitors have even been discovered, which are currently under investigation in clinical studies for their possible capacity to treat cancer, e.g., glioblastoma [99].

Protein p21 (CIP1) or cyclin-dependent kinase inhibitor 1A (*CDKN1A*) is another important tumor suppressor controlling cell cycle checkpoint mechanisms that inhibits cyclins, CDKs, or their complexes (cyclin-CDK2, cyclin-CDK1, and cyclin-CDK4,6) (Al Bitar and Gali-Muhtasib, 2019). Due to its effects, cell cycle progression is ultimately inhibited in several phases [100,101]. However, the pro-oncogenic function of p21 was also reported [102]. According to the other authors, the opposite role of p21 in carcinogenesis depends on cytoplasmic or nuclear p21 expression [103]. Recently, it has been suggested that p21 inducers might be valuable, especially in the treatment of cancers with downregulated p21 [104]. On the other hand, patients suffering from cancers with increased levels of p21 might be candidates for the treatment of p21 inhibitors. The expression of p21 is primarily regulated by p53, although p53-independent regulation exists [105].

Next, pRB is the protein associated with the development of retinoblastoma and many other cancers. This protein is encoded by the RB transcription corepressor gene 1 (*Rb1*), which was the first tumor suppressor gene discovered [106]. According to the literature, pRB can control the cell cycle via both canonical (E2F-dependent) and non-canonical (E2F-independent) mechanisms [107]. Canonical mechanisms are more common and depend on the phosphorylation status of the protein. The non-phosphorylated form of pRB binds and inhibits the transcription function of several members of the E2F family (E2F1, E2F2, E2F3), which results in suppression of the cell cycle progression and finally in the arrest of cell proliferation. Conversely, phosphorylation of pRB induced by cyclin-dependent kinases (mainly by CDK4/6) during G1/S phases disturbs pRB/E2F interaction allowing the transcription factor to fulfill its function to promote the cell cycle [108]. The non-canonical mechanism of cell cycle suppression involves increasing the levels of p27 protein, which is thought to be a CDK inhibitor. The expression and function of pRB are usually diminished by genetic [109] and epigenetic [110] inhibition, but also other regulating factors of the pRB pathway were determined. For example, oncoprotein from human papillomavirus HPV E7 can suppress the pRB-E2F interaction [111]. Additionally, repression of pRB occurs after several post-translational modifications [112]; for example, MDM2 targets pRB for degradation by the proteasome [113]. Furthermore, upstream regulators can be modified [107]. For example, genes for cyclins and CDKs can be amplificated or genes for cyclin-dependent kinase inhibitors can be deleted. As a result, phosphorylation and functional repression of pRB occur.

EA-induced upregulation of p53, p21, or both together has been demonstrated in several studies in various cancer cells. However, the molecular mechanisms underlying their overexpression and implications of this phenomenon are not yet fully elucidated. For instance, increased mRNA levels of p53 and p21 accompanied by reduced cell viability, G0/G1-phase arrest of the cell cycle, and apoptosis induction were found in case of exposure to EA in human bladder cancer cells (T24) [114]. Later investigations confirmed increased protein levels of p53 and p21 in other human bladder cancer cells (TSGH8301) treated with EA, which the authors attributed to the subsequent arrest of the cell cycle at the G0/G1 phase [60]. The next study has also documented the suppressive impact of EA on cell viability, in addition to its capacity to arrest the cell cycle at the G1 phase and induce the upregulation of protein expression for both p53 and p21 in two distinct ovarian carcinoma cells (ES-2 and PA-1) [56]. Other authors evaluated the antiproliferative effects of EA in human prostate cancer cell lines [115]. In this study, EA inhibited cell proliferation in the androgen-dependent cell line (LNCaP), whereas it had no impact on proliferation in the androgen-independent cells (PC-3 and DU145). Furthermore, the treatment of LNCaP cells with EA resulted in an upregulation of protein expression levels of several cell cycle regulatory proteins, including p21 and p27, which play a crucial role in regulating the transition of cells from the G1 phase to the S phase in the cell cycle. Thus, the authors have suggested that EA hindered the proliferation of prostate cancer cells by initiating both cell cycle arrest and apoptosis. In HepG2 cells, EA decreased cell viability and enhanced the expression of p53 and p21 [116]. In addition, these effects were further intensified when EA was combined with gamma irradiation. Later, it was observed that EA-treated HepG2 cancer cells exhibited elevated protein levels of p53, possibly contributing to apoptosis, which could be linked to an increase in ROS [117]. The recent research performed on EA-treated HepG2 cells and aimed at differentially expressed gene analysis declared the upregulation of *CDKN1A*, a gene encoding p21, as an essential mechanism responsible for cell growth inhibition via induction of cell cycle arrest at the G1 phase [77]. The significance of this discovery was affirmed via subsequent *CDKN1A* knockdown analysis. EA also had notable antiproliferative and apoptotic effects in rat C6 glioma cells, as well as the ability to increase protein expression of p53 [118,119]. The cell cycle arrest at the G0/G1 phase was another consequence of the action of EA in this cell line. In human breast cancer cells (MCF-7), EA inhibited cell proliferation and halted the cell cycle at the G0/G1 phase via an increase in p21 induced via overexpression of a signal molecule called mothers against decapentaplegic homolog 3 (Smad3) [68]. The antiproliferative and pro-apoptotic properties of EA were also demonstrated in human gastric adenocarcinoma cells (AGS), while one of the observed changes was a significant increase in mRNA expression of the p53 protein [120]. In another study using human cervical carcinoma cells (HeLa), EA significantly enhanced p53 and p21 only in combination with curcumin [121]. Nonetheless, a prior investigation involving various cervical carcinoma cells (CaSki) reported that EA induced an upregulation of p21 protein expression without a substantial elevation in p53 levels [122]. These findings suggest that EA could potentially induce G1 arrest and apoptosis via the upregulation of p21, employing a p53-independent mechanism. EA additionally potentiated the impact of quercetin in promoting the phosphorylation of p53 at Ser 15 and increasing p21 protein levels in the human leukemia cell line (MOLT-4), ultimately leading to apoptosis [123].

Additionally, in the study conducted by Mohammed Saleem and Selim [124] using different models of prostate cancer (LNCaP, p53^+/+^; 22RV1, p53^−/+^, and PC3, p53^−/−^), EA was reported as effective in reducing MDM2 at the gene and protein levels in all investigated cancer cell lines. These effects were also associated with an enhancement of protein expression of its downstream target p53 in LNCaP and 22RV1, but not in PC3 cells. Moreover, this change was not accompanied by a subsequent increase in MDM2 (a p53 target molecule) within an autoregulatory feedback loop. On the other hand, increased levels of p21 have been found in all prostate cancer cell lines, independent of the status of p53. This finding points to the ability of reduced MDM2 levels to exert p53-independent anticancer effects, which has been demonstrated by the downregulation of X-linked inhibitor of apoptosis protein (XIAP). However, most effects of EA observed in this study were primarily assessed in the context of apoptosis induction. Next, the authors revealed enhanced phosphorylation of p53 at ser15 and ser20 induced by EA in 22RV1 cells. It is noteworthy to mention that earlier research had elucidated that phosphorylation of p53 at ser20 diminishes the interaction between p53 and MDM2 [125]. Interestingly, urolithin A, a metabolite of EA, has also been shown to stabilize p53 [126] and attenuate MDM2-mediated p53 ubiquitination despite increased levels of MDM2 as a result of autoregulatory feedback loop [127]. In colorectal cancer, upregulation of p21 induced via specific microRNA is thought to be an important mechanism involved in anticancer properties (inhibition of cell growth, cell cycle arrest, and apoptosis) of ellagitannins [128]. However, some urolithins generated by gut microbiota were much more potent than EA in these effects.

Less is known about the effects of EA on pRB. As it was reported, EA exerted a decrease in phosphorylation of pRB and G0/G1 cell cycle arrest in MCF-7 breast cancer cells [68]. In addition, the results from the MTT assay confirmed decreased cancer cell proliferation. Finally, the authors highlighted that decreased pRB phosphorylation and effects on cell proliferation might be mediated by the activation of transforming growth factor β (TGF-β)/Smad3 signaling and subsequent overexpression of p21.

Equally important, several tumor suppressors may be non-functional at the same time in cancer cells. For example, the combined loss of *TP53* and *RB1* led to increased cell proliferation and diminished therapeutic outcomes for several androgen receptor antagonists [129]. Another essential point is the overlapping of both p53 and pRB signaling pathways via the function of p21, which can inhibit CD/CDKN complexes responsible for pRB phosphorylation [130]. Also, ubiquitination by MDM2 is common for both p53 and pRB proteins [112]. To sum up, EA may represent a promising potential drug, as it regulates the activity of all three major components of the p53–p21–pRB signaling pathway.

Furthermore, EA can also affect less important tumor suppressor proteins, such as PTEN (phosphatase and tensin homolog deleted on chromosome 10) that contribute to several aspects of cancer development and progression, including cell proliferation. Evidence has been provided in the literature that EA enhances the protein phosphatase activity of PTEN in melanoma cells (B16F10), resulting in reduced phosphorylation of focal adhesion kinase (FAK) [131]. Active phosphorylated FAK can stimulate cell cycle progression and proliferation signals, while its dephosphorylated form does not contribute to these processes. In this study, increased activity of PTEN also resulted in the accumulation of p53 as an indirect effect of EA. Figure 3 shows the findings described in this topic.

### 3.3. Ellagic Acid and Resisting Cell Death

The accompanying phenomenon of most malignant diseases is the deregulated response of tumor cells to proapoptotic stimuli. Therefore, the induction of cell death via apoptosis has been considered an essential mechanism of the action of cytotoxic drugs from the beginning of targeted research on antitumor chemotherapeutics, either directly or indirectly [132]. Under physiological conditions, apoptosis ensures normal tissue homeostasis regulation and a balance between cell proliferation and cell death [133,134]. This process is a response to cell damage caused by endogenous and exogenous stress factors such as DNA damage, endoplasmic reticulum stress, irradiation, UV radiation, heat shock, growth factor deficiency, viruses, Fas ligand (FasL), cytokines from the TNF family, and others [135,136].

In response to stress, the death machinery is activated via the intrinsic mitochondrial pathway of apoptosis, which is regulated by antiapoptotic and proapoptotic proteins from the Bcl-2 family. After mitochondrial membrane damage and permeabilization, cytochrome *c*, AIF, and other proapoptotic molecules are released, leading to the formation of the apoptosome and the activation of effector caspases. The extrinsic receptor-mediated pathway mediates signals from the extracellular space via ligands (TRAIL, TNFα, FasL) and death receptors (TNFR1, DR4, DR5), followed by the cleavage of initiator caspase 8 and effector caspases 3 and 7 [137]. Apoptosis can also be mediated by the significant tumor suppressor p53 after DNA damage or endoplasmic reticulum stress in a response known as the unfolded protein response (UPR) [138,139]. Morphologically, these complex molecular mechanisms result in chromatin condensation, nuclear fragmentation, cell shrinkage, cytoskeletal degradation, plasma membrane blebbing, and the formation of apoptotic bodies without eliciting an inflammatory response [140,141].

The disruption of some of these key regulatory mechanisms plays a major role in suppressing the apoptosis of tumor cells. Sublethal cell damage such as oxidative stress, hypoxia, DNA damage, starvation, and others, along with acquired genomic changes with oncogenic potential, lead to the development of drug-tolerant persistent or resistant cells. Therefore, it is necessary to explore additional forms of programmed cell death, such as necroptosis, ferroptosis, pyroptosis, and others, to complement apoptosis-inducing anti-tumor therapy [142,143]. Malignant cells with a reduced response to apoptotic stimuli or resistance to apoptosis are characterized by an imbalance in members of the Bcl-2 family, specifically overexpression of antiapoptotic proteins such as Bcl-2, Mcl-1, Bcl-xL, and/or underexpression of proapoptotic proteins such as Bax, Bad, Bim, and others. Reduced expression of death receptors and ligands, or expression of death receptors without death domains incapable of transmitting the stimulus into the cell, can disrupt the induction of the extrinsic pathway. In addition to increased expression of apoptosis inhibitors known as IAPs, decreased caspase expression, defects, and mutations in the tumor suppressor p53, which upregulates proapoptotic proteins and significantly influences the metabolism of tumor cells, are also involved in cell resistance to apoptosis [144,145]. Changes in the expression itself and the associated transcription of apoptosis-associated proteins, along with post-translational regulation changes, significantly affect not only the levels but also the function of expressed proteins. These processes not only hinder programmed cell death and contribute to the survival of malignant cells but can also accelerate tumorigenesis [146].

Currently, ongoing work is focused on new drugs and therapeutic approaches, such as cyclotherapy, which would be effective against tumor cells while sparing healthy non-tumor cells [147]. New drugs directly inducing apoptosis include Bcl-2 inhibitors, such as the FDA-approved venetoclax, Bcl-xL and Mcl-1 inhibitors, IAP inhibitors, Smac mimetics, PARP inhibitors, or other molecules in various stages of clinical trials, whose main mechanism of action is the induction of the extrinsic pathway of apoptosis [148,149,150,151,152].

**Figure 4:**
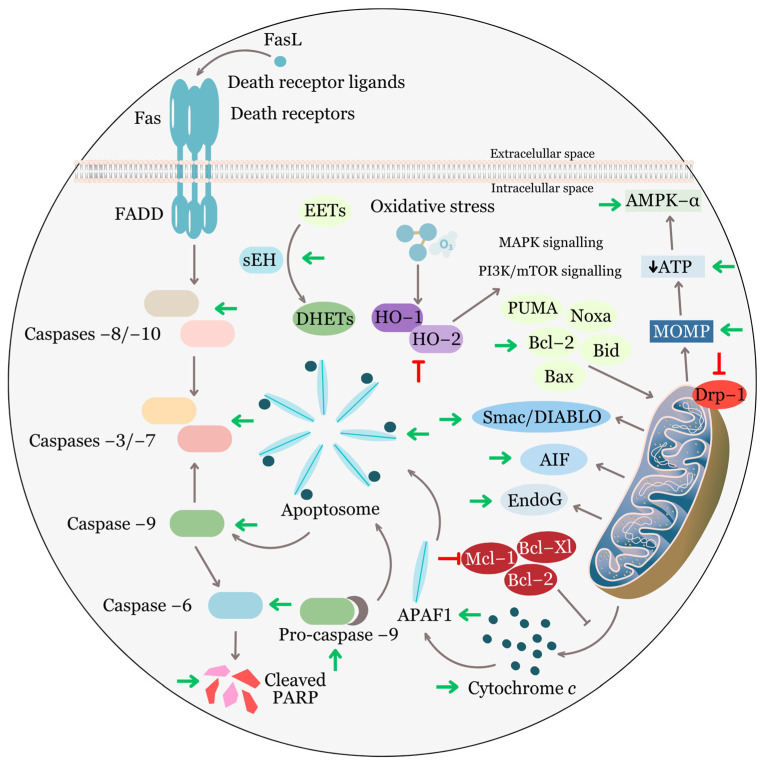
Effect of ellagic acid on intrinsic and extrinsic pathways of apoptosis. Upward-pointing green arrows signify heightened expression/activation, while red T bars indicate lowered expression or reduced activity.

Studies from recent years have shown that EA is capable of modulating several molecular targets, including those essential for the inhibition and induction of apoptosis (Figure 4). Along with influencing the activity of proteins regulating the cell cycle, such as p53, p21, cyclins, cyclin-dependent kinases, and their regulators, EA also affects members of the Bcl-2 family under in vitro conditions in human bladder cancer T24 cells and in vivo in glioblastoma U87 cells in xenografted Balb/c nude mice [59,114]. Reducing the ratio of levels between the antiapoptotic protein Bcl-2 and the proapoptotic protein Bax signifies a stimulus for cells that reduces their resistance to apoptosis and leads to cell death via the induction of the mitochondrial pathway of apoptosis [153]. This is due to changes in interactions between proapoptotic BH3-only proteins that inhibit Bcl-2, Mcl-1, Bcl-xL, and others. This activation leads to the oligomerization of Bax and Bak, allowing them to permeabilize the mitochondrial outer membrane [154]. Several studies confirm that EA significantly increases the Bax/Bcl-2 ratio with increasing concentration and exposure time and downregulates Bcl-xL and Mcl-1. EA also increases the expression of Bcl-2 inhibitory proapoptotic proteins PUMA and Noxa in prostate cancer cells [124]. This effect has been demonstrated in prostate cancer cell lines LNCaP (androgen-responsive prostate cancer), ES-2 and PA-1 (human ovarian carcinoma), cervical cancer HeLa cells, and non-small cell lung cancer A549 cells [56,61,115,155].

After the permeabilization of the mitochondrial outer membrane (MOMP), depolarization and loss of the transmembrane potential (Δψm) generated by proton pumps (Complexes I, III, and IV) occur. These are essential for oxidative phosphorylation and ATP production [156]. EA, via the upregulation of Bax and Bak, forms pores, significantly reducing the mitochondrial membrane potential (MMP) in the lung (HOP62 and H1975) and hepatocellular (HepG2) cancer cells. In these cells, ATP levels were significantly reduced [157,158,159]. A similar effect was observed in MCF-7 cells (human breast adenocarcinoma) after exposure to an extract from the plant *Terminalia chebula* from the *Combretaceae* family, which also contains EA as a significant component [160].

Following the induction of the intrinsic pathway of apoptosis by EA, in addition to the reduction in MMP, the release of cytochrome *c* into the cytosol occurs in pancreatic cancer cells MIA PaCa-2 and PANC-1 and colon adenocarcinoma cells HCT-15. Although EA does not directly affect mitochondria, it induces apoptosis via dose-dependent inhibition of the binding ability of the transcription factor NF-кB [74,158,161]. In addition to cytochrome *c*, proteins Smac/Diablo and Omi/HtrA2, and endonucleases apoptosis-inducing factor (AIF) and endonuclease G (EndoG) are released from the mitochondrial intermembrane space. Their role is oligonucleosomal DNA fragmentation [162,163]. Cytochrome *c* in a healthy cell shuttles electrons during oxidative phosphorylation from Complex III to Complex IV, but in the case of mitochondrial damage, it is one of the main inducers of cell death [164]. The mechanism of EA-induced caspase-dependent apoptosis involves the interaction of cytochrome *c* with APAF1 (Apoptotic protease activating factor 1), which is essential for the formation of the procaspase-9-activating heptameric protein complex known as the apoptosome. Ho et al. [60] studied the complex anti-proliferative and pro-apoptotic effect of EA on TSGH8301 (human bladder cancer) cells. They observed increased levels of apoptosis-inducing Bak, Bax, and Bid, along with mitochondrial membrane depolarization, induction of ROS production, and changes in intracellular calcium concentration, leading to increased levels of EndoG, Smac/DIABLO, AIF, cytochrome *c*, and APAF1 in the cytosol. In a study by Cheshomi et al. (2022) on gastric cancer cells AGS, in addition to upregulation of APAF1, significant increases in the expression of Bax, the tumor suppressor p53, and decreased expression of Bcl-2, NF-кB, and iNOS were observed after exposure to EA at concentrations of 15 and 30 µg/mL [120].

After proteolytic cleavage and activation of pro-caspase 9 through the apoptosome, active initiator caspase 9 is formed. It is capable of further activating effector caspases 3, 6, and 7 via intrachain activation cleavage [165]. Interestingly, in the presence of a mutant procaspase 9 that has lost its function, the apoptosome can directly activate caspase 3 [166]. The increase in caspase 9 activity in EA-treated pancreatic cancer cells PANC-1 and AsPC-1 subsequently led to increased activity and expression of one of the effector caspases, caspase 3 [167]. This effect is closely related to the modulation of some signaling pathways, such as PI3K/Akt, which was observed in colorectal cancer cells HCT-15 and lung cancer A549 cells [74,155]. Similar results were also reported by Edderkaoui et al., who, using a pan-caspase inhibitor, demonstrated that cell death induced by EA is caspase-dependent [161]. Suppression of cell proliferation, irreversible DNA damage, and activation of caspase 3 were also demonstrated in an animal model of prostate adenocarcinoma [115].

Unlike caspase activation cleavage, the cleavage of DNA repair enzymes and damage to DNA repair mechanisms stimulate the progression of cell death. Poly(ADP-ribose) polymerases (PARPs) are DNA-dependent nuclear enzymes that significantly impact cellular energetics and determine the fate of the cell. PARP1 stabilizes p53 and enhances its function, but under excessive damage, it can paradoxically facilitate the translocation of AIF into the nucleus, promote DNA fragmentation, and induce caspase-independent cell death known as parthanatos. To preserve the required levels of NAD+ and ATP and allow the cell to undergo programmed cell death, caspases cleave PARP into fragments, thereby inhibiting its activity [168,169]. In various p53-expressing ovarian cancer cells ES-2 and PA-1 and prostate cancer cells LNCaP, 22RV1, and PC3, a direct correlation between EA-induced caspase 3 activation and PARP cleavage in a dose-dependent manner (10–100 µmol/L) was demonstrated, culminating in a significant increase in the number of apoptotic cells [56,124]. Furthermore, EA sensitizes and reduces the resistance of breast cancer MCF-7 cells to apoptosis induced by γ-radiation [170], Ba/F3-insH cells (a murine interleukin-3 dependent pro-B cell line) treated with erlotinib [171], and HT-29 cells treated with 5-fluorouracil [172].

The fact that mitochondria are one of the main targets of EA’s anti-tumor action is also supported by the detection of additional mechanisms that contribute to mitochondrial dysfunction. Mitochondrial dynamin-related protein 1 (Drp-1) is a cytosolic guanosine triphosphatase (GTPase) responsible for the balance between mitochondrial division and fusion [173]. EA inhibits Drp-1-mediated mitochondrial fusion, contributing to the induction of the mitochondrial pathway of apoptosis in colorectal cancer (HCT116) and breast adenocarcinoma (MCF7) cells. This leads to a decrease in the respiratory capacity, loss of MMP, and cell cycle arrest [174]. Increased ATP production and intensified mitochondrial respiration are considered mechanisms that lead to the inhibition of cell death and increased resistance to anti-tumor treatment [175]. Results from a recent study show that EA reduced the expression of HIF-1α (Hypoxia-inducible factor-1α), a regulator of mitochondrial oxygen metabolism often overexpressed in cancer cells, while increasing phosphorylation and total levels of AMPK-α and ACC protein in HOP62 and H1975 lung cancer cells [157,176]. Changes in ATP-sensitive kinase AMPK regulation suggest that EA may also induce other mechanisms, such as autophagy and autophagy-associated cell death [177,178].

In addition to inducing the intrinsic mitochondrial apoptotic pathway mediated by Bcl-2 family proteins and the activation of effector caspases 3 and 6, and initiator caspase 9, Malik et al. also observed EA-mediated activation of caspase 8 in PC3 prostate cancer cells, which is considered essential for initiating the extrinsic, receptor-mediated pathway of apoptosis [179,180].

The machinery involving both the extrinsic and intrinsic apoptotic cascades further continues with morphological changes to cells and the formation of apoptotic bodies. One of these changes is the damage to the cell membrane and the flippase-mediated externalization of the phospholipid phosphatidylserine from the inner side of the plasma membrane to the outer side, which serves as an “eat me” signal for activated phagocytes. Such damaged membranes are permeable to other molecules such as propidium iodide (PI), 7-amino-actinomycin (7-AAD), and others [181,182]. Dual staining using a combination of the aforementioned components allows for quantifying the proapoptotic effect of various substances on cells. It was found that EA dose-dependently (10–40 µmol/L) induces apoptosis in cervical cancer cells (HeLa), gastric cancer cells (AGS), and colorectal cancer cells (HCT-116), increasing the population of Annexin V-positive, PI-positive, and double-positive cells compared to control cells [61,76,120]. This effect was observed in combination with EA and erlotinib [171]. Similar staining with acridine orange/propidium iodide (AO/PI) can be used to identify EA-induced apoptotic cells, which, in the presence of cell death, nuclear penetration, and nucleic acid intercalation, emit orange and red fluorescence instead of green fluorescence of live cells [56,160].

Another mechanism by which cells resist apoptosis is the induction of cytoprotective and anti-inflammatory proteins. Heme oxygenase-1 (HO-1) is an inducible enzyme capable of responding to oxidative stress, inhibiting the activity of proapoptotic K+ channels, and activating signaling pathways such as p38 MAPK and PI3K/Akt to protect cells from apoptosis [183]. It has been shown that EA significantly reduced the levels of two isoforms of this enzyme, HO-1, and HO-2, and increased the levels of sEH (Soluble epoxide hydrolase) in LnCap (prostate cancer cell line) cells [184]. sEH is the main metabolizer of epoxyeicosatrienoic acids (EETs), which can also contribute to the escape of cancer cells from apoptosis [185]. Other antiapoptotic proteins, such as RNA-binding protein HuR or sirtuins, with dual functions in carcinogenesis are often strongly expressed in cancer cells. Sirt1 can have antiapoptotic activity by deacetylating proapoptotic proteins and helping cells survive under genotoxic and oxidative stresses [186,187]. Vanella et al. [188] found that EA-induced apoptosis was associated with reduced expression of HuR and Sirt1, as well as decreased levels of transcription factors mTOR and β-catenin, with subsequent increased activity of caspase 3 and AIF.

A significant advantage of EA as a potential chemopreventive, anti-tumor, or adjuvant therapeutic agent in cancer treatment is its relative selectivity. In a study conducted by Salimi et al. (2015) [158] on CLL (chronic lymphocytic leukemia) B-lymphocytes and healthy B-lymphocytes, it was shown that EA significantly reduced the viability of cancer cells at a concentration of 10 µmol/L, while in healthy cells, this effect was observed only at a concentration of 200 µmol/L. Similarly, selective production of ROS, a decrease of MMP, mitochondrial swelling, cytochrome *c* release, and increased caspase 3 activity occurred in cancer cells [158].

### 3.4. Ellagic Acid and Deregulating Cellular Energetics

Cancer cells exhibit distinct energy requirements in comparison to normal cells, and these deviations are a hallmark of cancer that significantly contribute to tumor initiation, progression, and survival. One of the most widely recognized metabolic changes in cancer cells is known as the Warburg effect, wherein cancer cells predominantly rely on glycolysis, even when oxygen is available (aerobic glycolysis). This metabolic shift leads to increased glucose uptake and the production of lactate [189,190]. Additionally, cancer cells frequently undergo substantial modifications in their protein and lipid metabolism to support their rapid proliferation and growth. Apart from various metabolic and biochemical pathways, cancer cells hijack numerous signaling pathways to enhance metabolic flux through intermediary metabolism. These pathways, such as the PI3K/Akt/mTOR, AMPK, Wnt/β-Catenin, HIF, NF-κB, or MYC pathway, are often dysregulated, resulting in metabolic alterations that facilitate the growth and survival of cancer cells [191,192].

Despite the many unresolved questions in the realm of cancer metabolism, the pursuit of targeting it remains a highly promising avenue for creating more efficient cancer treatments while minimizing harm to healthy cells.

**Figure 5:**
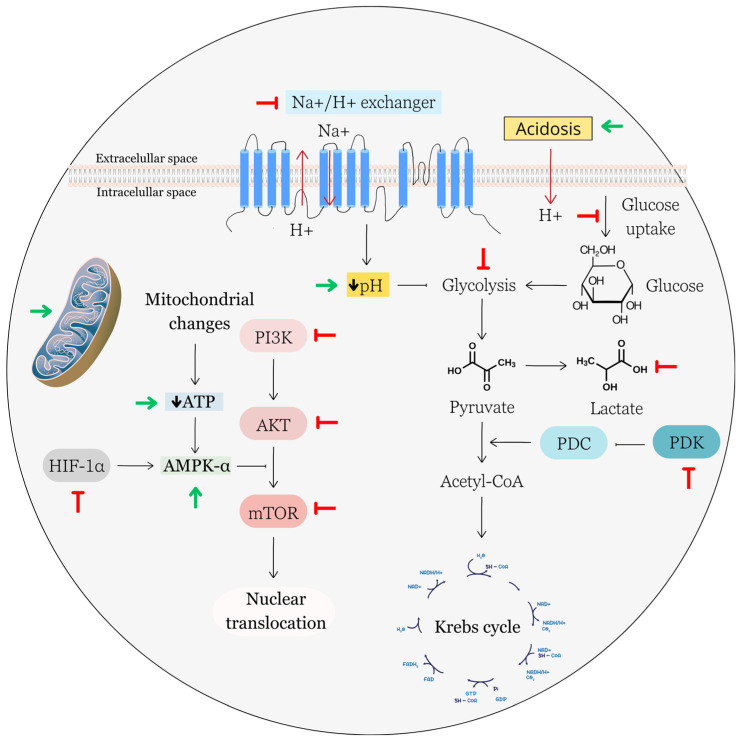
The effect of ellagic acid on selected metabolic pathways in cancer cells. Upward-pointing green arrows signify heightened expression/activation, while red T bars indicate lowered expression or reduced activity.

EA has demonstrated the potential to impact the metabolism of cancer cells via various mechanisms, showing promise in this regard (Figure 5).

The mammalian target of rapamycin (mTOR), also known as the mechanistic target of rapamycin, is a central regulator of cancer cell metabolism and plays a pivotal role in coordinating various cellular processes related to growth, proliferation, and energy utilization. mTOR exists in two main complexes, mTOR Complex 1 (mTORC1) and mTOR Complex 2 (mTORC2). Phosphorylation events are key for activating these complexes [193]. Furthermore, AMP-activated protein kinase (AMPK) is another important regulator that plays a central role in maintaining energy homeostasis and metabolic balance within cells. The relationship between AMPK and mTOR is intimately connected, with AMPK playing a pivotal role as a crucial inhibitory regulator of the mTOR pathway, primarily via mTORC1 [194]. Targeting the AMPK/mTOR pathway is an active area of research for developing cancer therapies that disrupt cancer cell metabolism and growth.

Ni and co-workers recently documented the pro-apoptotic effect of EA-treated colon cancer cells. They found that EA reduced mTOR phosphorylation together with AMP-activated protein kinase (AMPK) phosphorylation resulting in autophagy induction [177]. Another study showed that EA inhibited mTOR signaling in vitro as well as in vivo. Xia et al. (2020) [195] studied the antiproliferative and anticancer effect of EA on HeLa cells and on a cervical tumor-bearing BALB/c mouse model. In vitro, EA significantly decreased cancer cell proliferation, migration, and invasion, and this effect has been associated with decreased phosphorylation of the members of PI3K/AKT/mTOR/STAT3 signaling pathway. In vivo, EA suppressed cervical cancer growth and among other parameters, the authors documented decreased expression of phosphorylated form of mTOR detected via immunohistochemistry. Furthermore, a study conducted by Elsaid et al. [196] reported the antiproliferative and anti-invasive effects of EA associated with increased levels of phosphorylated AMPK in the SKOV-3 ovarian cancer cells. Moreover, EA prevented the phosphorylation of S6K1 and 4EBP, downstream targets of mTORC1 as well as Akt, indicating that inhibition of the Akt/mTOR pathway can also play a role in the antiproliferative effect of EA in ovarian cancer cells. Activation of AMPK in EA-treated lung cancer cells has also been documented by Duan et al. [157]. They reported activation of AMPK and, on the other hand, suppressed levels of HIF-1α. Given that HIF-1 activation in cancer cells induces substantial metabolic alterations that facilitate tumor growth and advancement (Infantino 2021), the potential for targeting the AMPK/HIF-1 pathway as a therapeutic approach in cancer treatment appears promising.

As it was mentioned above, aerobic glycolysis is the main source of energy for cancer cells. In a study carried out by Abdelazeem and colleagues in 2017, it was observed that EA had the capacity to regulate cytosolic pH by downregulating the expression of the Na^+^/H^+^ exchanger (NHE1) [197]. This led to intracellular acidification with subsequent impairment of glycolysis in endometrial carcinoma cells. In addition, this effect was also associated with a decrease in the cellular uptake of glucose and a notable reduction in lactate levels in supernatant. Another study showed the potential of EA to inhibit pyruvate dehydrogenase kinase (PDK), an enzyme that plays a key role in regulating the conversion of pyruvate to acetyl-CoA, a critical step in mitochondrial energy production. PDKs reduce the conversion of pyruvate to acetyl-CoA, favoring the metabolic pathway of glycolysis over oxidative phosphorylation. EA has been found to bind and inhibit PDK3, one of the four known isoforms of PDK [198]. 

As it was mentioned above, cancer cells undergo direct alterations in metabolic pathways, such as increased glycolysis or altered nutrient utilization. However, several indirect regulatory mechanisms also contribute to cancer cell-associated metabolic changes.

It is well known that the tumor suppressor gene TP53, which encodes the protein p53, plays a central role in maintaining genomic stability and preventing the development of cancer [199]. In addition to these activities, TP53 also regulates metabolic pathways involved in glycolysis, or oxidative phosphorylation [200]. However, frequent TP53 mutations result in divergent effects on metabolic reprogramming between wild-type (wt) p53 and gain-of-function (GOF) mutant p53. While wt p53 generally inhibits glycolysis, GOF mutant p53 has been noted to promote glycolysis in cancer cells [201], and the restoration of p53’s metabolic functions appears to be one of the potential options for anticancer therapy. Several experimental studies have showed a close relationship between the antiproliferative effect of EA and the activation of p53 [121,124,131]. While the predominant effects of p53 induction via EA have been reported to involve cell cycle arrest and the triggering of apoptosis, it remains a possibility that p53-induced alterations in cancer cell metabolism could also play a role in the antiproliferative effects of EA.

Mutations or amplifications in oncogenes, such as Myc, can directly or indirectly promote changes in metabolism. Myc, for example, regulates the expression of genes involved in glycolysis, glutamine metabolism, and nucleotide biosynthesis [202]. In a study conducted by Lim et al. [203], EA has been shown to influence extracellular acidosis, a metabolic characteristic of cancer tissue. This modulation of extracellular acidosis by EA has been linked to alterations in cancer cell behaviors, particularly reduced invasiveness. Furthermore, this effect has been associated with the downregulation of cancer-related genes, including COX1, COX2, snail, twist1, and c-Myc.

### 3.5. Ellagic Acid and Enabling Replicative Immortality

Continuous cell proliferation or tumor cell immortality of malignant cells is promoted by the reactivation of a ribonucleoprotein enzyme called telomerase (terminal transferase), which prevents the shortening of telomeres during repeated replications [204]. It is well known that telomeres, repetitive (TTAGGG) DNA-protein complexes at the 3′ ends of linear chromosomes, prevent genome instability, and conversely, their shortening leads to the cell growth arrest, replicative cellular senescence, and aging of the organism [205]. On the other hand, the maintenance of their length by telomerase is crucial for carcinogenesis [206]. Increased expression of this enzyme is characteristic of the majority of cancer cells, while most normal human somatic cells lack it [207]. In humans, telomerase is composed of a catalytic protein subunit known as human telomerase reverse transcriptase (hTERT), encoded by the *hTERT* gene, and an RNA component named human telomerase RNA (hTR) or human telomerase RNA component (hTERC), encoded by the *hTERC* gene [206]. It has been suggested that reactivation of telomerase occurs primarily via increased expression of its key catalytic subunit hTERT [208].

In addition to regulating telomere length (canonical function), telomerase and hTERT perform many other functions such as impact on proliferation (due to Wnt/β-catenin pathway or NF-kB and p65 pathway), cell survival, apoptotic resistance, adhesion, migration, stem cell renewal, inflammation, chromatin configuration and DNA repair, mitochondrial DNA protection and resistance to ionizing radiation (non-canonical functions) [209,210]. Thus, telomerase and hTERT were identified as potential therapeutic targets for cancer treatment [211].

Few studies have focused on EA and factors influencing replicative immortality. An older study showed that *Phyllanthus urinaria* extract containing several bioactive phytochemicals, including EA, might dose-dependently inhibit telomerase activity, as well as the mRNA expression level of hTERT and human telomerase-associated protein 1 in nasopharyngeal cancer cells [212]. On the other hand, it has been demonstrated that EA might have a dual effect on hTERT. When used alone, it upregulated hTERT alpha + beta + mRNA levels in estrogen receptor-positive MCF-7 breast cancer cells but significantly decreased upregulated level of hTERT induced by hormonal growth stimulator (17beta-estradiol) [213]. Finally, a huge study aimed at molecular modeling analyses of several phytochemicals from *Acacia nilotica* identified EA as a potential inhibitor of hTERT [214].

Interestingly, hTERT activity may also be downregulated by the increased level of p53 (Chen et al., 2017). Other authors reported that p53 can downregulate various genes needed for telomere metabolism [215]. The ability of EA to upregulate p53 has been already described in the previous section of the article.

### 3.6. Ellagic Acid and Inducing Angiogenesis

In 1971, Dr. Judah Folkman published a basic paper that laid the foundation for the field of angiogenesis research [216]. As tumors grow, the existing blood supply may become insufficient to meet their demands. Angiogenesis enables tumors to establish new blood vessels, ensuring a constant supply of nutrients, oxygen, and other essential factors to cells, which supports their expansion. This phenomenon plays a crucial role in the growth, progression, and metastasis of cancer [217]. Cancer cells and other cells within the tumor microenvironment (TME) release signaling molecules that stimulate the growth of new blood vessels. The TME influences angiogenesis via various mechanisms including secretion of angiogenic factors, hypoxia, interaction with immune cells, or extracellular matrix remodeling [218,219]. Therefore, regulating angiogenesis could potentially serve as an effective approach to treating malignant tumors. Nowadays, there are numerous antiangiogenic drugs in clinical practice including drugs targeted to vascular endothelial growth factor (VEGF) and its receptors (e.g., bevacizumab, ramucirumab, aflibercept), several receptor tyrosine kinases (e.g., apatinib, sunitinib, sorafenib, levantinib), hypoxia-inducible factor (e.g., belzutifan), receptor fusion proteins (e.g., Ziv-afibercept), or immunomodulatory agents with anti-angiogenic effects (e.g., lenalidomide, thalidomide) [220,221,222].

Notably, the VEGF and its corresponding receptor (VEGFR-2) hold significant importance as therapeutic targets for addressing diverse cancer types [223].

Moreover, several natural compounds have been reported to modulate angiogenesis [224,225,226].

**Figure 6:**
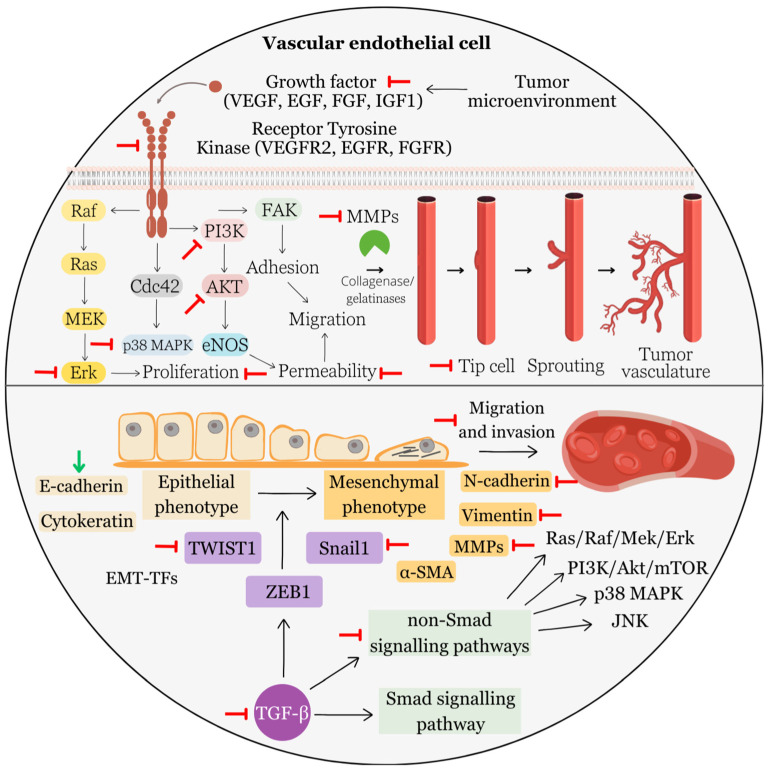
Ellagic acid’s impact on angiogenesis, cancer cell migration, and invasiveness. Upward-pointing green arrows signify heightened expression/activation, while red T bars indicate lowered expression or reduced activity.

EA has been studied for its potential anti-cancer properties, including its ability to inhibit angiogenesis. The mechanisms via which EA may inhibit angiogenesis involve its interactions with various molecular pathways and factors involved in blood vessel formation (Figure 6).

In a study by Losso et al. [227], it was observed that EA hindered the proliferation of human umbilical vein endothelial cells (HUVECs) and impeded the formation of tube-like structures by these cells. Furthermore, cancer cells treated with EA demonstrated a notable reduction in the secretion of matrix metalloproteinase (MMP)-2 and MMP-9. Additionally, the release of vascular endothelial growth factor (VEGF) was suppressed in cancer cells treated with EA. The ability of EA to inhibit MMP-2 activity and secretion has been confirmed by Huang et al. [228] in EA-treated HUVECs. Moreover, they also documented in vivo antiangiogenic effect of EA using CAM assay. At concentrations of 5 and 10 μM, EA led to a reduction in the angiogenic index by 62% and 83%, respectively. Another study showed that EA effectively suppresses the phosphorylation of VEGFR-2 induced by VEGF in endothelial cells (ECs). Additionally, EA inhibits the phosphorylation of platelet-derived growth factor receptors (PDGFR) in smooth muscle cells, which inhibits the downstream signaling pathways of these receptors. Moreover, EA inhibits the migration and tube-like structure formation of VEGF-treated ECs [229]. Later, in the study conducted by Vanella et al. [184], EA has been reported to decline activity/expression of different angiogenic factors in prostate cancer cells including heme oxygenase (HO), a potent modulator of cell growth and angiogenesis, VEGF, fibroblast growth factor (FGF), granulocyte colony-stimulating factor (G-CSF), and hepatocyte growth factor (HGF) expression. Recently, Kisacam [230] documented the dose-dependent effect of EA on some angiogenic factors in the human intestinal cancer cell line. At lower concentrations (10 and 20 μM), EA led to a substantial increase in VEGF levels. However, at higher doses (40 and 100 μM), a notable reduction in VEGF levels was observed in EA-treated cancer cells. Conversely, with the exception of exposure to 40 μM EA, there was a significant decrease in HO protein levels in Caco-2 cells as compared to the control group. The interference of EA with angiogenesis was also clearly demonstrated in a study by Wang and co-workers [231]. The antiangiogenic potential of EA was thoroughly examined via investigations conducted in vitro, ex vivo, and in vivo settings. In vitro experiments revealed that EA markedly decreased VEGF-induced EC proliferation, migration, and the formation of tube-like structures. These effects were linked to the inhibition of VEGFR-2 tyrosine kinase activity, along with the suppression of downstream signaling pathways in EC like MAPK and PI3K/Akt. Ex vivo assessments demonstrated that EA notably diminished the length and density of EC sprouts around chick aortic rings after stimulation with VEGF. Furthermore, the antiangiogenic effect of EA has also been confirmed in vivo using CAM assay and a breast cancer xenograft model. A detailed study showed that EA has the capability to establish hydrogen bonds and aromatic interactions within the ATP-binding domain of the VEGFR-2 kinase unit and thus markedly modulate several VEGF-dependent angiogenesis processes. Furthermore, in a study conducted by Ceci et al. [232], EA exhibited a reduction in VEGFR-2 expression in human bladder cancer cells, with varying degrees of inhibition ranging from 39% to 72% depending on the cell line analyzed. Surprisingly, EA did not affect the VEGF-A-induced phosphorylation of VEGFR-2. In another study, EA was highlighted as a potent inhibitor of angiogenesis in a pancreatic cancer cell xenograft model conducted on Balb C nude mice [57]. Administration of EA to the experimental animals resulted in a decrease in the expression of various angiogenesis markers, including COX-2, HIF1α, VEGF, and VEGFR, alongside other pro-angiogenic molecules such as IL-6, IL-8, MMP-2, and MMP-9. Immunohistochemical analysis of the tumor tissue demonstrated a notable reduction in the formation of new blood vessels in EA-treated animals. On the other hand, the findings reported by Pitchacarn et al. [233] indicated that EA treatment did not induce significant alterations in the secretions of MMP-2 and MMP-9 from prostate cancer cells. Interestingly, they found that EA significantly suppressed the activity of these gelatinases. Based on this observation, the authors hypothesized that EA has the potential to diminish the invasive attributes of prostate cancer cells by targeting protease activity. Regarding matrix metalloproteinase, studies showed that MMPs are known to require zinc ions for their enzymatic activity, and this zinc-dependent activation is a key aspect of their function [234]. Huang et al. [235] conducted a study to explore whether the antiangiogenic effect of EA is associated with MMP-2 inhibition and if this inhibition could be counteracted by zinc supplementation. They found that EA-induced suppression of the MMP-2 activity and secretion was reversed by the zinc addition. Based on these results, the authors suggest that the zinc-chelation properties of EA play an important role in the antiangiogenic effect via MMP inhibition.

In summary, EA has emerged as a promising natural compound with the potential to target cancer angiogenesis, a critical process that fuels tumor growth, invasion, and metastasis.

### 3.7. Ellagic Acid and Activating Invasion and Metastasis

Tumor infiltration and metastasis remain the most challenging hallmarks of cancer and are responsible for the majority of cancer-related fatalities [236]. Despite being the best predictor of poor prognosis, it is still inadequately understood, with a substantial portion of its molecular mechanisms primarily elucidated from experiments using cancer cell lines [237]. The metastasis of tumor cells, often described as the invasion–metastasis cascade, encompasses a series of stages. These stages include initial local invasion into the neighboring stroma, followed by intravasation into the bloodstream, withstanding shear stress, extravasation to distant locations, and finally, adaptation to new microenvironments. This progression culminates in the emergence of micro-metastases, which subsequently evolve into discernible secondary tumors [238]. Numerous signaling pathways contribute to the invasive and metastatic properties of cancer cells. The epithelial–mesenchymal transition (EMT) process, for instance, facilitates the conversion of stationary epithelial cells into motile mesenchymal cells, promoting invasion and dissemination [239]. Several key molecular players, including transcription factors such as Snail, Slug, and Twist, orchestrate this transition [240]. Moreover, growth factors such as TGFβ and EGF stimulate the invasive behavior of cancer cells and are implicated in various stages of the metastatic process [241]. Tumor invasion requires intricate remodeling of the extracellular matrix (ECM) to enable cancer cells to breach physical barriers. Matrix metalloproteinases (MMPs) represent a family of Ca^2+^-dependent Zn^2+^-containing endopeptidases capable of breaking down proteins in the extracellular matrix, thereby facilitating cancer cell migration, invasion, and metastasis [242]. Understanding the molecular basis of invasion and metastasis opens up avenues for targeted therapies. Emerging treatments that target EMT, ECM remodeling, and specific signaling pathways show promise in limiting the spread of metastasis.

Recent studies have suggested that EA may play a multifaceted role in inhibiting invasion and metastasis (Figure 6). EA has been shown to influence the expression of various genes and proteins involved in cellular adhesion, migration, and invasion. It downregulates factors that promote invasion while upregulating those that inhibit it, thus creating an unfavorable environment for cancer cells seeking to metastasize. Several studies have demonstrated that EA may exert inhibitory effects on MMPs, specifically on MMP-2 and MMP-9. Among the numerous MMPs identified (over 20 in total), MMP2 and MMP9 have been linked to the aggressive behavior of cancer [243]. By downregulating these MMPs, EA may play a crucial role in impeding the degradation of the ECM and suppressing cancer cell migration and invasion [203,244,245,246]. Furthermore, a study conducted by Pitchakarn et al. (2013) revealed that EA had a significant impact on reducing the enzymatic activity of collagenase/gelatinase produced by the rat prostate cancer cell line (PLS-10). Additionally, EA exhibited concentration-dependent inhibition of collagenase IV activity. These findings suggest that EA possesses the capacity to hinder the metastasis and invasion of prostate cancer cells by targeting protease activity [233].

Another study showed that EA exhibited a dose-dependent inhibition of the growth, migration, and invasion of pancreatic cancer cells (PANC-1). The effect was potentially mediated by upregulating E-cadherin and downregulating vimentin, consequently impeding the progression of EMT [58]. During EMT, the pivotal protein E-cadherin in epithelial cells is downregulated, while vimentin, a significant mesenchymal protein in interstitial cells, undergoes upregulation [247]. In the study conducted by Kim et al. [167], it was demonstrated that EA inhibited both of these processes in pancreatic cancer cells (PANC-1, AsPC-1). The inhibition of proliferation was explained by an increase in apoptosis through the caspase-3 and caspase-9 pathways, a consequence of EA’s action. The suppression of migration was associated with an upregulation of E-cadherin and decreased levels of TGF-β, MMP-2, and MMP-9. Notably, these effects were only observed in PANC-1 and AsPC-1 cells, not in MIA PaCA-2 cells. This implied that the impact of EA on pancreatic cancer was influenced by cell characteristics. Additionally, a study by Wang et al. (2017) revealed that EA suppressed the metastasis of glioblastoma cells (U87 and U118) by controlling the expression of EMT-related transcription factors and MMPs. EA inhibited glioblastoma invasion in the U87 xenograft model by upregulating E-cadherin and downregulating the expression of transcription factors, such as Snail, MMP-2, and MMP-9 [59]. The results of a study by Lim et al. (2019) suggested that EA suppressed acidity-enhanced migration and invasion of gastric cancer cells by inhibiting the expression of multiple factors (COX1, COX2, snail, twist1, and c-myc). Due to this factor, it may be a promising candidate for treating cancer in the presence of acidosis. Extracellular acidosis, a key metabolic factor in tumor tissue, is a significant metastatic factor in many types of cancer cells [248,249,250]. It alters the expression of oncogenes and the aforementioned genes linked to EMT (*SNAIL1*, *TWIST1*, *MYC*). Under acidic conditions, gastric cancer cells become more invasive, with elevated expression of MMP-7 and MMP-9. In concentrations below 10 μM, EA effectively suppressed MMP-7 and MMP-9 expression triggered by acidity, restraining the migratory and matrigel-infiltrating capacity of gastric cancer cells [203].

By specifically targeting and reducing the expression of TGF-β1, FSCN1 (Fascin Actin-Bundling Protein 1), and vimentin, EA exhibited the potential to obstruct pivotal processes closely associated with the development and progression of hepatocellular carcinoma (HCC). TGF-β1 assumes a critical role in HCC development by instigating the EMT process [245]. Meanwhile, FSCN1 plays a pivotal role in cell invasion and migration, both essential aspects of cancer metastasis. Upon activation of TGF-β1, FSCN1 experiences overexpression in HCC, thereby further exacerbating the progression of EMT. Moreover, vimentin acts as a significant marker during EMT, with heightened levels observed in cells undergoing this transformation, thereby contributing to the aggressive nature of cancer cells [251,252]. Furthermore, intervention with EA inhibited the migration, invasion, and proliferation of melanoma cells (WM115 and A375) by reducing the activation of EGFR [72]. EGFR is typically strongly activated in various tumorigenic processes. It also significantly contributes to the progression and metastasis of melanoma [253]. Elevated EGFR expression in tumor cells enhances cell adhesion to the extracellular matrix and facilitates distant metastasis [254]. EGFR has emerged as a prognostic marker in various cancer types, indicating a poor prognosis. Consequently, the administration of EA presents a promising adjunctive therapeutic approach for melanoma patients [72].

In another study, it was reported that EA effectively inhibited the proliferation, migration, and invasion of anaplastic thyroid cancer cells (ATC) by suppressing the Wnt/β-catenin and PI3K/Akt pathways [255]. Abnormal activation of these signaling pathways collectively correlates with cancer progression and contributes to the promotion of EMT via epigenetic control [256]. Regarding the PI3K pathway, the concurrent administration of EA and the PI3K inhibitor GDC-0941 to various breast cancer cell lines resulted in a significant reduction in cell proliferation, migration, and in vitro invasion. This combination also led to a decreased tumor initiation rate and a reduction in metastasis formation in vivo [257]. Other authors demonstrated that EA could effectively inhibit the invasion and migration of endometrial carcinoma cell lines KLE and AN3CA by suppressing PI3K phosphorylation and downregulating the expression of MMP9. Additionally, EA was observed to inhibit lung metastasis in BALB/c nude mice, which were intravenously injected with KLE and AN3CA cells. Collectively, these findings strongly suggest that EA possesses potent antitumor properties against endometrial cancer, effectively suppressing cell invasion and migration by targeting the PI3K signaling pathway both in vitro and in vivo [66].

Moreover, EA effectively impeded the growth and metastasis of breast cancer by directly targeting ACTN4 (Alpha-actinin-4). This intervention was concomitant with a reduction in the population of cancer stem cells. Cancer stem cells expressing ACTN4 displayed heightened capabilities, including increased mammosphere formation and enhanced tumorigenesis in vivo [258]. Increased ACTN4 expression is directly correlated with more advanced cancer stages, elevated incidence of metastasis, poor patient prognosis, and tumor chemoresistance [259]. Silencing of ACTN4 by EA led to the inhibition of malignant cell proliferation, formation of cell colonies, and improved suppression of metastatic potential. Taken together, these findings suggest that ACTN4 could potentially serve as a critical target for EA treatment, particularly in the context of breast cancer stem cell-related metastasis [258].

### 3.8. Ellagic Acid and Avoiding Immune Destruction

The immune system plays an important role in carcinogenesis. Several suppressive mechanisms are involved in the control and elimination of mutated malignant cells. However, cancer cells influenced by humoral and cellular components of TME can escape from the control and promote cancer development. To eliminate these negative inhibitory mechanisms and reintroduce an effective malignant cell-clearing activity of immunocompetent cells, several immunotherapeutic approaches were proposed [260].

The acute inflammatory process is a part of the defense mechanisms. However, chronic inflammation, which affects many cellular pathways, can lead to the initiation and development of different forms of cancer [261]. Inflammatory cells produce a number of growth factors, cytokines, ROS, proteases, matrix metalloproteinases, and a large scale of other enzymes. They can activate transcriptional factors and bring about cellular proliferation, genomic instability, angiogenesis, resistance to apoptosis, invasion, and metastasis [262]. Cells and their products form part of the TME and ensure dynamic interactions between tumor cells and the TME and modulate response to immunotherapy. Some of those inflammatory molecules allow cancer cells to proliferate, survive, and metastasize [263]. On the other hand, there exist molecules that can influence inflammatory parameters and exert an anti-carcinogenic potential by different mechanisms. For example, some cytokines have shown antitumor activity by influencing tumor antigen presentation, T-cell priming and activation, T-cell infiltration, and cancer cell death via stimulation of the adaptive and innate cell immunity [264,265]. All these data indicate that inflammation and related factors can play a dual role in the initiation and progression of the malignant process, modulating immune system processes and their interaction with tumor cells in the TME.

The discovery of checkpoint inhibitory proteins, e.g., CTLA-4, PD-1, PD-L1, and the development of antibodies that block their reducing immunologic responses resulted in one of the most promising therapeutic modalities in anticancer immunotherapy. However, despite considerable clinical progress, a number of interacting factors within the TME can influence the therapeutic response to immunotherapies [266]. Moreover, a limited number of patients can benefit from checkpoint inhibition treatment due to the lack of appropriate initial indication markers, development of resistance, or occurrence of severe immunity overstimulating adverse reactions. These negative effects result in intensive need and reasonable search for subsequent therapeutic combinatorial modalities, which include the use of naturally occurring substances.

EA exerts potent anticarcinogenic effects by several mechanisms, including inhibition of tumor cell proliferation, induction of tumor cell apoptosis and autophagy, break of DNA binding to carcinogens, blocking virus infection, disturbing inflammation, angiogenesis, inhibition of tumor cell metastasis and invasion, affection of tumor metabolic reprogramming and drug-resistance processes [261,267]. It is also characterized by its antioxidant properties and ability to influence the production of pro-inflammatory mediators (e.g., TNF-α, IL-1β, IL-6) resulting in anti-inflammatory effects (see below) tightly related to carcinogenesis. Please note that the role of EA in the modulation of anticancer immunity reactions seems to be also important. All those properties together with above mentioned malignant process suppressing mechanisms make EA a highly interesting potential anticancer compound [268].

The effects of EA on immunity parameters have been studied in several in vivo experiments and on different immune cells in in vitro conditions. An early study investigating the effect of EA on common immunological surveillance was performed in B6C3F1 mice (a hybrid strain extensively used in carcinogenicity bioassays) exposed continuously to EA in drinking water at 0.5, 1.0, or 2.0 mg/kg/day for 28 days. Among several different parameters, responses of natural killer (NK) cell activity, cytotoxic T lymphocyte activity, and IgM antibody plaque-forming cells were investigated. Obtained data have shown that subchronic exposure to EA (2.0 mg/kg) caused significant suppression of specific IgM antibody responses in the treated group and suppressed cytotoxic T-cell function in the 0.5 and 1.0 mg/kg EA-treated groups. All other immunological parameters were within normal ranges [269].

Additional immunomodulating effects of EA in different subpopulations of immunocompetent cells were discovered and described in the following experimental studies. The results of these experiments were summarized in a review by Rahimi et al. [270]. It has been found that the influence of EA on immune cell subpopulations results in strong anti-inflammatory effects. For example, EA in phytohemagglutinin stimulated human peripheral blood mononuclear cells (PBMC) resulting in decreased cell proliferation, reduced levels of IL-13 and TNF-α, and increased IL-2 production [271]. In the same cell population, EA antagonized the stimulatory effect of macrophage migration inhibitory factor and inhibited NF-κB nuclear translocation and chemotaxis [272]. Similar immunomodulatory effects with inflammation-suppressing activity were observed in PE regularly containing EA as an active constituent. In this case, the extract was used to influence neutrophil activity stimulated by FN-formyl-methionyl-Leucyl-phenylalanine and phorbol myristate acetate, inhibition of myeloperoxidase activity and decreased ROS level were noted [273]. The functions of human basophilic cell line KU812 (chronic myelogenous leukemia) stimulated by phorbol-12-myristate 13-acetate plus calcium ionophore A23187 were also affected by PE. The effect consisted of the inhibition of pro-inflammatory cytokines IL-6 and IL-8 production and NF-κB gene expression [274].

In another study, the authors investigated some additional immunological responses to EA. The experiments studying the effects of EA were performed on the lipopolysaccharide (LPS)-induced bone marrow-derived dendritic cells. The results have shown that EA suppressed LPS-induced expression of co-stimulatory molecules (CD80 and CD86). However, the expression of major histocompatibility complex (MHC) class I and class II were not suppressed. These reactions were mediated via the EA-induced block of LPS-induced c-Jun N-terminal kinase (JNK) activation. At the same time, LPS-mediated expression of proinflammatory cytokines (IL-12 and IFN-γ) was diminished by EA. This was confirmed by the concurrent use of a JNK inhibitor, which antagonized EA effects on the levels of IL-12 and IFN-γ. It has been suggested that EA demonstrated promising immunomodulatory effects in this preclinical model through the regulation of dendritic cell maturation and suppression of humoral immunity [275].

Taken together, these studies show that EA is capable of downmodulating pro-inflammatory mediators and stimulating the production of anti-inflammatory cytokines [268]. Decreasing inflammatory and aforementioned interfering immunity reactions EA may interfere with carcinogenic processes and suppress initiation and development of malignant cell transformation and progression.

On the other hand, immune regulatory mechanisms could have a dual effect on carcinogenesis. Progression in tumoral growth, invasion, and metastasis formation is among the other factors attributed to tumor cell escape from immunity system control. This control is effectively diminished via overexpression of immune checkpoint proteins and their receptors, mainly PD-1, PD-L1, and CTLA-4, among others. A restart of immunocompetent cell activities in recognition and subsequent elimination of cancer cells are believed to participate in the effective inhibition of tumoral growth and the overall spread of invasive malignant cells.

The following experimental study advanced toward the evaluation of EA effects on PD-L1 expression in human urinary bladder cell lines. This research was performed because of FDA approval of atezolizumab (humanized monoclonal antibody against the immune checkpoint PD-L1) for platinum-treated advanced urothelial cancer. Analysis of UM-UC-3 (hypertriploid epithelial human bladder cells) and T24 (transitional cell human bladder carcinoma) cells showed that EA downregulated the expression of the immune checkpoint PD-L1 in tumor cells. It was suggested that the effect of EA might potentially enhance the efficacy of anti-PD-L1 treatment and contribute to a decrease in immunosuppressive mechanisms that favor disease progression [232].

Another more recent study evaluated the effects of *Rubus coreanus* extract containing a large amount of EA on cellular PD-1/PD-L1 blocking activity. *Rubus coreanus* extract and EA dose-dependently inhibited PD-1/PD-L1 binding in PD-1/NFAT Jurkat (Jurkat recombinant cell line containing a firefly luciferase gene under the control of the nuclear factor of activated T cells response element) and PD-L1/aAPC CHO-K1 (cells stably expressing human PD-L1 and a cell surface protein designed to activate cognate T-cell receptors in an antigen-independent manner) cell co-cultures. These results were confirmed in in vivo experiments in humanized PD-1 mice bearing MC38 colorectal tumor. The efficacy of oral application of *Rubus coreanus* extract was comparable to PD-1 antibody activity [276].

**Figure 7:**
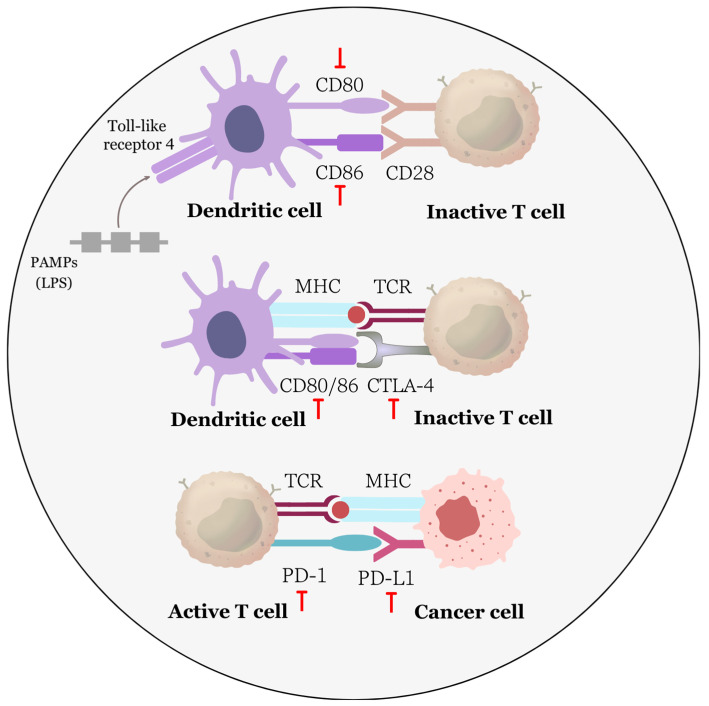
Effect of ellagic acid on specific aspects of the immune system. Red T bars indicate lowered expression or reduced activity.

The involvement of EA and its beneficial effect in anti-cancer immunomodulation was demonstrated as well in the evolution of anti-PD-1/PD-L1 resistance in murine and human lung tumors. The mechanism of resistance resulted from increased collagen levels in the tumor extracellular matrix which induced T cell exhaustion via the leukocyte-associated immunoglobulin-like receptor 1 (LAIR1). EA was found to decrease tumor collagen deposition through lysyl oxidase-like protein-2 (LOXL2) suppression. Additionally, a reduction in tumor growth was observed when EA was combined with anti-PD-L1 treatment, showing its effectiveness [277]. Figure 7 shows the findings described in this topic.

### 3.9. Ellagic Acid and Tumor-Promoting Inflammation

Oxidative stress, inflammation, and the associated constant production of pro-inflammatory cytokines, inflammatory mediators, growth factors, and prostaglandins produced by activated macrophages lead to the development of chronic inflammation. Inflammation is the body’s response to cell damage accompanied by massive production of inflammatory cytokines (interleukins and tumor necrosis factor (TNF-α), transcription factors (IFN-γ), and nuclear factor (NF-κB) [278]. Prolonged chronic inflammation, subsequent genetic mutations, and cellular differentiation can instigate a precancerous state that ultimately culminates in the development of cancer. Statistically, it was evaluated that approximately 15% of cancer diseases are associated with chronic inflammation [279]. Factors from the external environment as well as infection and autoimmune diseases influence the development of chronic inflammation. The most well-known examples of chronic inflammation that can lead to cancer are inflammatory bowel disease, chronic hepatitis, Helicobacter pylori-induced gastritis, pneumonia caused by inhaling tobacco smoke, inflammation associated with obesity, hyperglycemia, excessive accumulation of lipids, or bacterial products [280]. Due to the interplay between chronic inflammation and cancer, targeting chronic inflammation represents a promising avenue for cancer prevention.

As it was mentioned above, EA is an excellent antioxidant with the ability to capture free radicals and ROS, thereby protecting cells from oxidative stress, inhibiting the mutagenicity of carcinogens, and at the same time stimulating the immune system to produce anticancer molecules [281]. EA has also been shown to have very strong anti-inflammatory effects. Experimental works inspired by the results of the antioxidant and anti-inflammatory effects of EA currently support the idea of its chemopreventive and antitumor effects [11,282]. The anti-inflammatory effects of EA in relation to the inhibition of carcinogenesis have been demonstrated by numerous in vivo and in vitro studies.

Several results show that natural substances suppressing NF-κB or products regulated by NF-κB should have potential in the prevention and treatment of cancer [283]. From the results of the work by Khan et al. [284], it is evident that EA interacts with the Rel homologous domain of NF-κB. This interaction affects the expression of genes encoding pro-inflammatory molecules, resulting in a significant reduction in the levels of pro-inflammatory cytokines and chemokines. Another study focused on the effect of EA on inflammatory responses in pancreatic cancer cells, showed suppression of the NF-κB activity in EA-treated cancer cells. Furthermore, it was reported that EA triggered the loss of mitochondrial membrane potential and the activation of mitochondrial apoptotic pathways in these cells [161]. Additionally, in a study conducted by Cheng et al. [58], EA has been reported to suppress the proliferation of human pancreatic carcinoma PANC-1 cells both in vitro and in vivo. In vitro, EA inhibited the proliferation, migration, and invasion of PANC-1 cells. In animal experiments, EA significantly reduced tumor growth and prolonged the survival of experimental animals. Western blot analyses showed that these effects have been associated with decreased expression of pro-inflammatory markers COX-2 and NF-κB. Another group of researchers conducted a study examining the impact of EA on colon cancer induced by 1,2-dimethylhydrazine in rats. They observed heightened expressions of pro-inflammatory proteins and cytokines in 1,2-dimethylhydrazine-treated rats. However, oral administration of EA led to a significant reduction in NF-κB levels, subsequently resulting in a decrease in inflammatory markers, including iNOS, COX-2, TNF-α, and IL-6 [285]. In addition, treatment of the human gastric adenocarcinoma cell line (AGS) with EA reduced cell proliferation and migration. Moreover, EA treatment led to alterations in the expression of genes involved in inflammation including, iNOS, NF-κB, IL-8, and TNF-α [120]. In another study, EA exhibited a substantial increase in both the expression and functionality of antioxidant enzymes, such as catalase, superoxide dismutase, and glutathione reductase, in the livers of mice bearing Dalton lymphoma. Furthermore, EA downregulated protein kinase C expression and prevented the activation of NF-κB. This outcome can be attributed to the fact that PKC is activated by ROS. Consequently, the heightened antioxidant defense induced by EA inhibited PKC activation, subsequently resulting in the suppression of NF-κB activation [286]. A significant study of the effect of EA on HepG2 hepatocellular carcinoma cells after irradiation demonstrated that EA stimulated the formation of ROS, and increased the expression of p53 and p21 proteins as well as markers of apoptosis (Bax and caspase-3). At the same time, EA reduced the levels of survival markers as well as the inflammatory markers TNF-α and IL-6 [116].

Additionally, numerous studies have centered on pomegranate as a significant source of EA. For instance, a study conducted by Bishayee et al. in 2013 explored the impact of PE on inflammation in diethylnitrosamine (DENA)-induced liver carcinogenesis in rats [287]. Notably, DENA alone led to a significant increase in the expression of various inflammatory markers, including iNOS, 3-nitrotyrosine, COX-2, and NF-κB. However, when PE was administered preventively, it exhibited a remarkable decrease in the expression of all these inflammatory markers. According to the authors’ suggestions, the modulation of the NF-κB signaling pathway could play a pivotal role in the anticancer effects observed with the use of PE. In a separate investigation, the ingestion of PE was found to inhibit chemically induced lung carcinogenesis. Among the multitude of identified proteins, PE notably repressed inflammatory markers such as CD31, iNOS, and NF-κB [288].

**Figure 8:**
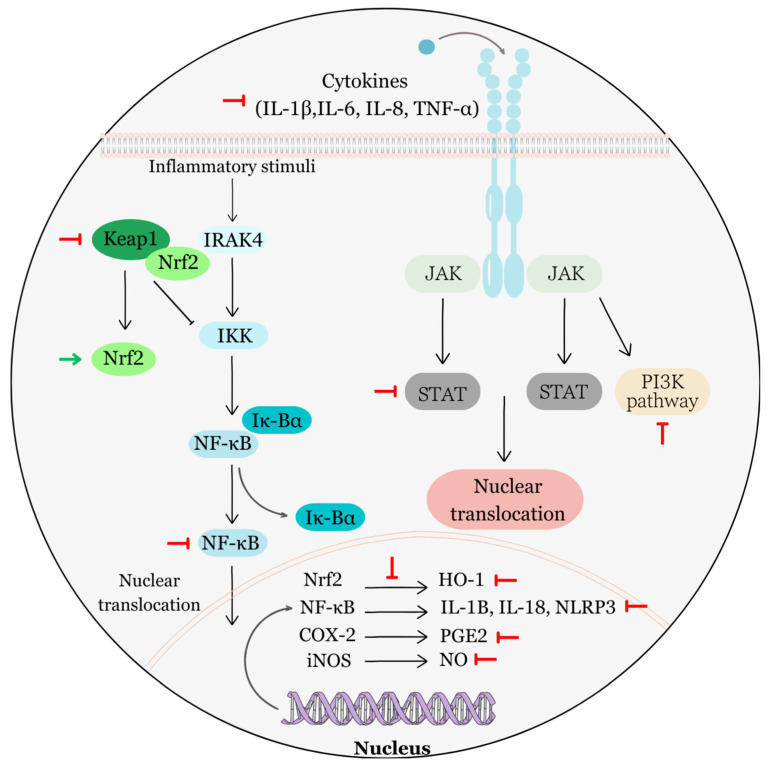
Anti-inflammatory effect of ellagic acid. Green arrows indicate increased expression/activation, while red T bars indicate lowered expression or reduced activity.

The analysis of the level of secreted pro-inflammatory cytokines/chemokines by prostate tumor cells DU145 and PC3 cells was devoted to the work of Wang et al. [289]. Luminex Multiplex analysis revealed that pro-inflammatory cytokines/chemokines such as IL-6, IL-12p40, IL-1β, and RANTES were reduced by PE with high content of EA. Therefore, they believe that EA has the potential to reduce inflammation and influence cancer progression. Similar results were also achieved by Rettig et al. [290] and Wang et al. [289] when PE reduced the levels of pro-inflammatory markers TNF-α and NF-κB. Subsequently, Mandal et al. (2017) [291] investigated the anti-inflammatory mechanism of PE in a rat mammary carcinogenesis model induced by dimethylbenz(a)anthracene. Their findings indicated that PE exhibited a significant anti-inflammatory effect, as evidenced by the reduced expression/activity of several proteins involved in inflammatory processes, including COX-2, HSP90, and NF-κB. Conversely, PE increased the expression and nuclear localization of Nrf2, a transcription factor that plays a pivotal role in safeguarding cells against inflammation and oxidative stress [291]. In summary, Figure 8 shows the actions of EA targeting inflammation.

### 3.10. Ellagic Acid and Genome Instability and Mutation

Cells undergo a continuous cycle of damage and repair. DNA damage arises from various endogenous and exogenous sources, such as such as radiation [292], chemical mutagens [293], DNA repair deficiencies [294], oxidative stress-inducing high ROS levels [295], viral infections and chronic inflammation [296], cell-cell fusion [297] and others. Moreover, telomere shortening can lead to next-genomic instability and promote cancer development [298]. DNA repair pathways aim to correct these errors and damages, ensuring the preservation of the genetic code and preventing the accumulation of mutations that could lead to diseases such as cancer. Key players in this intricate mechanism are the Ataxia Telangiectasia Mutated (ATM) and RAD3-related (ATR) kinases [299,300].

A few years ago, five priority targets against genomic instability were identified: prevention of DNA damage, promotion of DNA repair mechanisms, restoration of deficient DNA repair; impairing centrosome clustering, and inhibition of telomerase activity [301].

In line with these, an attenuation of genome instability is relevant in the context of chemoprevention used to prevent the initiation or progression of cancer. The main goal of chemopreventive agents is to reduce the genetic alterations that can lead to carcinogenesis.

In a study conducted by Aiyer et al. [302], EA was reported to prevent oxidative stress-induced DNA damage. Co-incubation of 4-hydroxy-17ß-estradiol E2 (4E2) and CuCl_2_ with EA significantly decreased levels of 8-oxodeoxyguosine (8-oxodG), a marker of oxidative DNA damage compared to 4E2/CuCl_2_ only. Moreover, EA significantly decreased levels of oxidative adducts in vivo. In addition, EA has been found to upregulate DNA repair genes, including xeroderma pigmentosum group A complementing protein (XPA), DNA excision repair protein (ERCC5), and DNA ligase III (DNL3). Later, Berni and co-workers [303] demonstrated a protective effect of EA in an N-methyl-N’-nitro-N-nitrosoguanidine (MNNG) model of genotoxicity. MNNG is a well-known carcinogen targeting primarily guanine resulting in the formation of O^6^-methylguanine adducts [304]. The outcomes of their study indicated that EA effectively decreased the micronuclei formation both in vitro and in vivo. However, this reduction was observed only when EA was administered before exposure to MNNG. Other authors documented the protective effect of EA against benzo[a]pyrene (BaP)-induced DNA adducts. BaP is widely recognized as a potent carcinogen and mutagen. When metabolized into benzo[a]pyrene-7,8-diol-9,10-epoxide, it can bind to DNA, leading to the creation of DNA adducts. These adducts can introduce mistakes during DNA replication and repair processes, ultimately elevating the risk of cancer [305]. In a study conducted by Zahin et al. [306], EA significantly decreased number of BaP-induced DNA adducts. Moreover, using different salmonella strains in the Ames test, EA significantly decreased the number of mutations induced by several mutagens including sodium azide, methyl methanesulfonate, BaP, and 2-aminoflourine. Additionally, at the tested concentrations ranging from 50 to 500 μM, EA displayed no mutagenic effects. A similar protective effect of EA in BaP-induced genotoxicity has also been confirmed by Varshney and co-workers [307] in both in vitro (Ames test) and in vivo (mouse bone marrow micronucleus) assays. Administration of EA led to a decrease in the frequency of BaP-induced revertants in the *S. typhimurium* strain TA100. Furthermore, administration of EA resulted in a reduction in the average number of BaP-induced polychromatic erythrocytes in the micronucleus test in mice. Additionally, Western blotting results indicated a downregulation of the cytochrome P450 (1A1) isoform, which in turn prevented the activation of benzo[α]pyrene. In another study, the protective effect of EA against acrylamide-induced genotoxicity in human lymphocytes has been studied [308]. This study found that exposure to acrylamide at a concentration of 50 μM resulted in increased production of reactive oxygen species (ROS), elevated lipid peroxidation, disruption of the mitochondrial membrane potential, lysosomal damage, and DNA damage. Conversely, co-administration of EA at concentrations of 25 and 50 μM, among others, suppressed acrylamide-induced DNA damage as evidenced by a reduction in the levels of 8-hydroxy-2′-deoxyguanosine, a marker of oxidative stress-induced DNA damage.

Furthermore, a recent research analysis performed on rat embryonic fibroblasts [309] demonstrated that EA acid can restore telomerase activity inhibited by exposure to phosalone, a common agricultural organophosphate pesticide capable of ROS induction [310] and genotoxicity [311].

Interestingly, EA exhibited statistically significant upregulation of sirtuin 6 at the protein level in Caco2 cells [312]. This member of the sirtuin family is a multifaced protein that contributes to the maintenance of telomere function, DNA integrity, and genome stability [313].

Furthermore, multiple studies have established the antimutagenic and antigenotoxic properties of EA when exposed to various mutagenic or genotoxic substances. These include benzene [314], cyclophosphamide [315], nicotine [316], and aflatoxin B1 [317], as well as sodium azide and N-methyl-N’-nitro-N-nitrosoguanidine [318].

Additionally, in a study conducted by Matić et al. [319], the results demonstrated that EA did not exhibit genotoxic effects when tested on somatic and germ cells of *Drosophila melanogaster*.

However, it is important to mention that several in vitro studies also reported an increase in DNA damage or increased ROS formation under EA exposure in cancer cells. As an example, EA promoted DNA damage and ROS generation in the TSGH8301 human bladder cancer cell line [60]. In addition, significantly increased EA-induced DNA damage was confirmed in human glioblastoma cell lines U87 and U118 by determining the presence of the phosphorylated form of H2AX histone variant (γ-H2AX), which serves as a molecular marker of DNA damage [59]. In some studies, the administration of EA led to the induction of PARP-cleavage in pancreatic cancer cells (PANC-1) [57] or prostate cancer cell lines [124]. PARP cleavage serves as a marker of apoptosis, but the cleavage of PARP has also functional implications [168]. The cleaved PARP fragments lose their ability to perform their roles in DNA repair, further promoting the apoptotic process.

In summary, the dual impact of EA on DNA damage and ROS production underscores the need for comprehensive research to elucidate the precise mechanisms governing these processes. Understanding the context in which ellagic acid promotes DNA protection or induces damage is vital for harnessing its potential benefits effectively, particularly in cancer therapy and prevention.

Molecular and cellular mechanisms of EA in relation to cancer hallmarks are presented in Table 1.

## 4. Conclusions

The experimental evidence presented in this review underscores the promising role of EA as a potential therapeutic agent in addressing the fundamental hallmarks of cancer. Our analysis reveals that EA exhibits multifaceted actions, impacting key aspects of cancer biology making it a compelling subject for future investigations and the development of innovative strategies to enhance cancer prevention and therapy. While the experimental evidence presented here is promising, it is crucial to acknowledge the complexity of cancer as a disease and the need for further research, including clinical trials, to validate the efficacy and safety of EA-based therapies. Additionally, considerations such as bioavailability and optimal dosing regimens should be explored in greater depth.

## Figures and Tables

**Table 1:** Molecular mechanisms of ellagic acid in influencing cancer hallmarks. Arrows indicate an increase (↑) or decrease (↓) in the levels/activity of the molecules.

Cancer Hallmark	Mechanism of Action	References
*Sustaining Proliferative Signaling and Evading Growth Suppressor*	Suppression of cell proliferation, S, G1, and G2/M phase cell cycle arrest, inhibition of signaling pathways, estrogen antagonism, ↑ p53, p21, p15, PTEN ↓ cyclin D1, CDK2, CDK4, CDK6, COX-2, IL-1β, IL-6, IL-8, NF-κB, Notch pathway, PI3K/Akt pathway, Hedgehog pathway, Akt pathway, Wee1, JAK/STAT3 pathway, EGFR, PIK3CA, PIK3R1, TGF-β/Smad3 signaling, β-catenin, axin 1, axin 2, Myc, survivin, K-ras, MCM2-7, phospho-pRb, PKCα, PKCβ, and PKCγ, MDM2, XIAP, FAK	[56,57,58,59,60,61,62,65,66,68,69,71,73,74,75,76,77,78,124,125,131]
*Resisting Cell Death*	Induction of mitochondrial and extrinsic pathway of apoptosis, permeabilization of the mitochondrial outer membrane, DNA damage and fragmentation, inhibition of mitochondrial respiration, and mitochondrial damage ↑ Bax, Bak, Bid, PUMA, Noxa, cytochrome *c*, EndoG, Smac/DIABLO, AIF and APAF1 release, the activity of caspases-3, -8, -9, phosphorylation and expression of AMPK-α and ACC, PS externalization ↓ Bcl-2, Bcl-xL, Mcl-1, MMP, Drp-1, HIF-1α, HO-1, HO-2, sEH, HuR, Sirt	[56,59,60,74,114,120,124,153,157,158,159,161,167,174,176,179,180,184,188]
*Deregulating Cellular Energetics*	Inhibition of aerobic glycolysis, inhibition of Akt/mTOR signaling pathway ↑ phospho-AMPK, intracellular acidification ↓ phospho-mTOR, phospho-S6K1, 4EBP, HNE1, PDK, acetyl-CoA	[177,196,197,198]
*Enabling Replicative Immortality*	Inhibition of telomerase activity, modulation of hTERT activity	[212,213,214]
*Inducing Angiogenesis*	Inhibition of angiogenesis, inhibition of formation tube-like structures of endothelial cells and migration, reduction of angiogenic index, inhibition of VEGF signaling pathways ↓ MMP-2, MMP-9, VEGF secretion, phospho-VEGFR-2, phospho-PDGFR, HO activity, MAPK signaling, PI3K/Akt signaling	[184,227,228,229,231,232,233]
*Activating Invasion and Metastasis*	Impeding the degradation of the ECM, suppression of cell migration and invasion, inhibition of epithelial-mesenchymal transition ↑ E-cadherin ↓ enzymatic activity of collagenase/gelatinase, vimentin, TGF-β, MMP-2, MMP-9, Snail, COX1, COX2, c-Myc, Twist1, FSCN1, Wnt/β-catenin pathway, PI3K/Akt pathway, ACTN4	[59,167,203,233,244,245,246,255,258]
*Avoiding Immune Destruction*	Antioxidant activity, modulation of the production of pro-inflammatory mediators, inhibition of PD-1/PD-L1 binding ↑ NK cells response, cytotoxic T lymphocyte activity, IL-2, effectiveness of anti-PD-L1 treatment ↓ IL-13, TNF-α, macrophage migration inhibitory factor, NF-κB nuclear translocation, ROS production, myeloperoxidase activity, expression of CD80 and CD86, JNK activation, IL-12, IFN-γ, PD-L1, LOXL2, collagen deposition	[232,268,269,271,272,273,275,277]
*Tumor-Promoting Inflammation*	ROS scavenging, oxidative stress protection, inhibition of mutagenicity of carcinogens, anti-inflammatory effect, reduction in the levels of pro-inflammatory cytokines and chemokines ↑ catalase, superoxide dismutase, glutathione reductase ↓ NF-κB, COX-2, iNOS, TNF-α, IL-6, IL-8, PKC, 3-nitrotyrosine, CD31, IL-12p40, IL-1β, RANTES, HSP90, Nrf2	[58,120,281,284,285,286,287,288,291]
*Genome Instability and Mutation*	Prevention of oxidative stress-induced DNA damage, upregulation of DNA repair genes, micronuclei formation ↑ XPA, ERCC5, DNL3, restore telomerase activity, Sirt6 ↓ 8-oxodG, cytochrome P450 (1A1)	[302,304,306,307,308,309,312]
